# Exploring the associations between the biomechanical and psychological mechanistic pathways of lower back pain development amongst persons with lower-limb amputation: A study protocol

**DOI:** 10.1371/journal.pone.0314523

**Published:** 2025-02-06

**Authors:** Alexandra Withey, Dario Cazzola, Abby Tabor, Elena Seminati

**Affiliations:** 1 Department for Health, University of Bath, Bath, United Kingdom; 2 Faculty of Health and Applied Sciences, University of the West of England, Bristol, United Kingdom; Universiti Sains Malaysia, MALAYSIA

## Abstract

**Introduction:**

The global rise in lower-limb amputations is mainly due to diabetes and vascular complications. Amputations cause serious physical and psycho-social disabilities which impair locomotion and compromise patients quality of life. Biopsychosocial factors such as altered gait parameters, poor prosthetic fit, depression, fear avoidance behaviours and stigma increase the risk of individuals incurring lower back pain; the leading cause of secondary disability amongst persons with lower-limb amputation. Exploring the potential mechanistic pathways of lower back pain development is important to inform timely patient-centred programmes of care. Currently, limited information exists to inform the design of such programmes. Thus, there is a pressing need to understand the bio-behavioural, psychological, and social features of individuals with lower-limb amputation in the presence of lower back pain.

**Methods and analysis:**

This proposed study protocol employs a prospective longitudinal study design that aims to explore the determinants of lower back pain amongst 30 adults with unilateral lower-limb amputation over 12-months. Biomechanical gait variables, trunk and lower-limb muscle activations, and objective pain measurements will be monitored every 3-months, and their relationship will be investigated. This information can be used to explore the characteristics of lower back pain and will inform future care management and rehabilitation processes. A data repository will be created and will be accessible through the University of Bath library website (https://library.bath.ac.uk/home).

**Trial reference number:**

The study is registered at IRAS ID: 321729 and at ClinicalTrials.gov: NCT06243549.

## Introduction

Lower-limb amputations cause serious physical and psycho-social disabilities that can compromise an individual’s quality of life and necessitate diverse prosthetic solutions (e.g. artificial limbs) to increase mobility [1]. However, locomotion post-amputation is altered. Gait parameters reported to change in persons with unilateral lower-limb amputation (LLA) include altered joint load distribution caused by gait asymmetry and dangerous compensations, poor prosthetic fit or alignment, and increased sound limb contact time [2]. These biomechanical factors increase the risk of long-term complications, including lower back pain (LBP), sound limb knee osteoarthritis (KOA) [3, 4], and osteoporosis in the bones of the amputated leg. Persons with LLA spend less time on their amputated limb [5], leading to osteopenia and subsequent osteoporosis due to insufficient loading of the bones, and overloading-induced KOA in the joints of sound limb. Although these long-terms complications can be related (e.g. pain in the joints can affect gait), in this protocol we will focus specifically on LBP.

### Epidemiology of limb amputations

The number of people with limb loss is growing steadily. Globally, the incidence and prevalence number of people living with traumatic amputation increased from 11.37 million and 370.25 million (16.4% rise) in 1990, to 13.23 million and 552.45 million (49.2% rise) in 2019 [6]. The global annual incidence of individuals living with diabetes-related LLA is approximately 94.82 cases per 100,000 individuals with diabetes between 2010–2020 [7]. Public Health England (2019) statistics show a 14% rise in amputations between 2015–2018 compared to 2012–2015. This is due to increased life expectancy and a corresponding higher incidence of diabetes and vascular diseases [8, 9]. The UK has approximately 55,000–60,000 individuals with limb loss [10], 86% of which are LLA [11]. Transtibial (below knee-level) amputations (TTA) account for approximately 31% of LLA and transfemoral (above knee-level) amputations (TFA) account for 24% [12]. Approximately 65–90% of LLAs are attributed to vascular problems, and ~5% are caused by trauma, malignancy, and congenital limb deficiencies [12].

### Prevalence of lower back pain amongst persons with lower-limb amputation

LBP is a serious global health problem and is considered the leading cause of disability worldwide, with 11–38% of the general population experiencing LBP over a one-year duration [13–15]. The annual cost of LBP in the United States is approximately $100 billion, with two-thirds attributed to socio-economic factors including reduced productivity [16]. LBP is an important cause of secondary disability amongst persons with LLA [17], with national annual prevalence rates in four countries (United Kingdom, United States, Sweden, and Vietnam) between 50–90% [18–23]. Following amputation, a one-month prevalence rate of 20–30% [24, 25] and a two-year prevalence of 60% [22] have been reported. Approximately 25–50% of individuals with LBP report the condition as ‘bothersome’ and experience difficulties performing activities of daily living (ADL), including ambulating and standing up from a chair [18].

### Lower back pain characteristics and gait amongst persons with lower-limb amputation

The experience of pain is a complex biopsychosocial problem, however the interplay between biological, psychological, and social stressors, which may play a role in pain development, is poorly characterised amongst both individuals with and without LLA [26, 27]. Persons with LLA often experience different types of pain, including residual limb pain (RLP), phantom limb pain (PLP), joint pain, and LBP, which can limit individuals from achieving ADLs and pursuing meaningful goals. RLP occurs in the remaining part of the amputated limb often attributed to physical peripheral factors (e.g. local wound healing, neuromas, or poor socket fitting). PLP is defined as a sensation relating to a limb or organ which has been amputated [23, 28], involving peripheral and central nervous system adaptations. LBP is defined as pain around the lumbar spine, it can be multidimensional, and is described by pain location, intensity, frequency, and its impact on ADLs [23]. The experience of frequent (daily or weekly) LBP amongst persons with LLA is associated with moderate/severe physical disability and difficulties performing ADLs [18, 21, 22]. Common examinations for LBP diagnosis and monitoring are radiology, validated questionnaires, and functional outcome measures [29].

Although numerous studies have assessed persons with LLA walking ability [30–33], few have investigated the experience of pain as a variable that may affect walking performance and discrepancies exist regarding the influence of pain on gait outcomes. Currently, there are no longitudinal studies investigating when LBP starts and the consequences, and key variables which may be associated with LBP, including psycho-social factors and muscle activations, are rarely reported. Furthermore, the contributory role of gait analysis factors, such as increased ground reaction forces (GRF), joint loads, and greater time spent on the sound limb [4, 34], have been heavily debated [26]. This is due to studies inaccurately reporting pain location and few specified pain frequency, severity, or intensity [26]. Additionally, others used pain questionnaires which have not been validated in the literature [26].

Previous research has found an association between prosthetic use and the prevalence of LBP amongst persons with traumatic LLA [35, 36]. As persons with traumatic LLA are generally younger and more active compared to individuals with vascular LLA [26], greater prosthetic use amongst this population may predispose individuals to LBP. Research has proposed that the severity of LBP is greater with more proximal amputation locations [23, 37–39]. However, discrepancies exist within the literature regarding the accuracy of these findings [26]. When studying LBP prevalence amongst persons with LLA, it is important to account for one’s activity levels, time spent active on the prosthesis, the number of years of prosthetic use, and psycho-social factors, such as anxiety and depression [26].

The association between amputation and pain is unclear due to the multifactorial nature of each condition. In addition, variables such as age, pre-existing pain, and clinical characteristics of people with limb loss are rarely taken into account, and with that, small sample sizes typically engaged in this research domain makes inferences greatly limited for specific patient groups. There is evidence that LBP is more bothersome than PLP and RLP [23], and is associated with shorter walking distances one-year post amputation [40, 41]. Risk factors for LBP include type and level of amputation and prosthesis, poor prosthetic fit, gait modifications, limb-length discrepancies, and psycho-social factors such as fear avoidance and depression [42].

### Psycho-social risk factors for lower back pain amongst persons with lower-limb amputation

Although psycho-social factors do not appear to be predictive of the initial onset of LBP [29], factors such as anxiety, depression, and fear avoidance have been associated with an increased risk of chronic LBP development in both individuals with and without LLA [43, 44]. A national survey amongst persons with LLA found that depression was significantly associated with ‘bothersome’ LBP [19]. Depression is more common amongst persons with LLA compared to the general population [45, 46], therefore depressive moods may present a greater risk for LBP [47].

Persons with LLA who display fear of using the prosthetic limb may develop dysfunctional movement patterns and subsequent LBP [47–49]. Chou and Shekelle [50] reported that higher fear-avoidance scores were associated with worse functional outcomes and a greater risk of LBP development at 3-, 6-, and 12-months. However, due to the small body of literature, the potential contributory role of psycho-social risk factors to LBP development requires further attention [51]. Acute pre-amputation pain may increase the likelihood of individuals displaying post-operative fear of movement behaviour, which in turn may lead to pain chronification, disability, and reduced patient function [52]. Pre-amputation pain has been associated with a greater risk of post-operative pain 3- and 6-months post-amputation, and a greater duration (>1 month) and intensity of pre-amputation pain is a risk factor for chronic pain [53–55].

### Objectives and hypotheses

To address this gap, the proposed study protocol will explore the bio-behavioural, psychological, and social characteristics of LBP amongst persons with unilateral LLA over a period of 12-months after their amputation. The whole spectrum of LBP will be explored, including both acute and chronic LBP. To date, this has not been explored in the current literature, and is therefore a novelty and strength of the proposed protocol. Specifically, interactions between bio-behavioural factors of movement (i.e. gait characteristics, trunk and lower-limb muscle activations), and the psycho-social aspects of pain and function will be investigated. Here, the term ‘function’ refers to the comprehensive overview of each participant’s everyday interactions and long-term targets, defined by the participant in terms of person-specific activities and goals. Consideration of these variables aim to improve an understanding grounded in patient-specific function, helping to decipher the relationships between the biopsychosocial characteristics of LBP in individuals with LLA. In doing so, this investigation looks to inform tailored care management and rehabilitation processes.

We hypothesise that individuals who display fear avoidance behaviours will have altered joint range of motion, which in turn will influence muscle activation patterns, leading to greater loading on one side and asymmetries between the sound and residual limb, and therefore increase the risk of LBP development.

Hypothesis 1 (HA1): Gait characteristics will change with time post-amputation and will stabilise following a reduction in movement pattern and trunk muscle activation asymmetries.
Hypothesis 2 (HA2): Individuals with a greater risk factor pattern of stressors will experience more pain following the amputation.
Hypothesis 3 (HA3): Persons with LLA who experience different types of pain will be at a greater risk of reduced functionality over time.
Hypothesis 4 (HA4): Greater scores on psycho-social assessment of pain questionnaires, asymmetric gait patterns, greater and asynchronous activation of trunk muscles, and changes in spatiotemporal characteristics of gait, such as slower gait speed, will be associated with altered pain and function.

## Methods and analysis

### Study design

**Fig 1:**
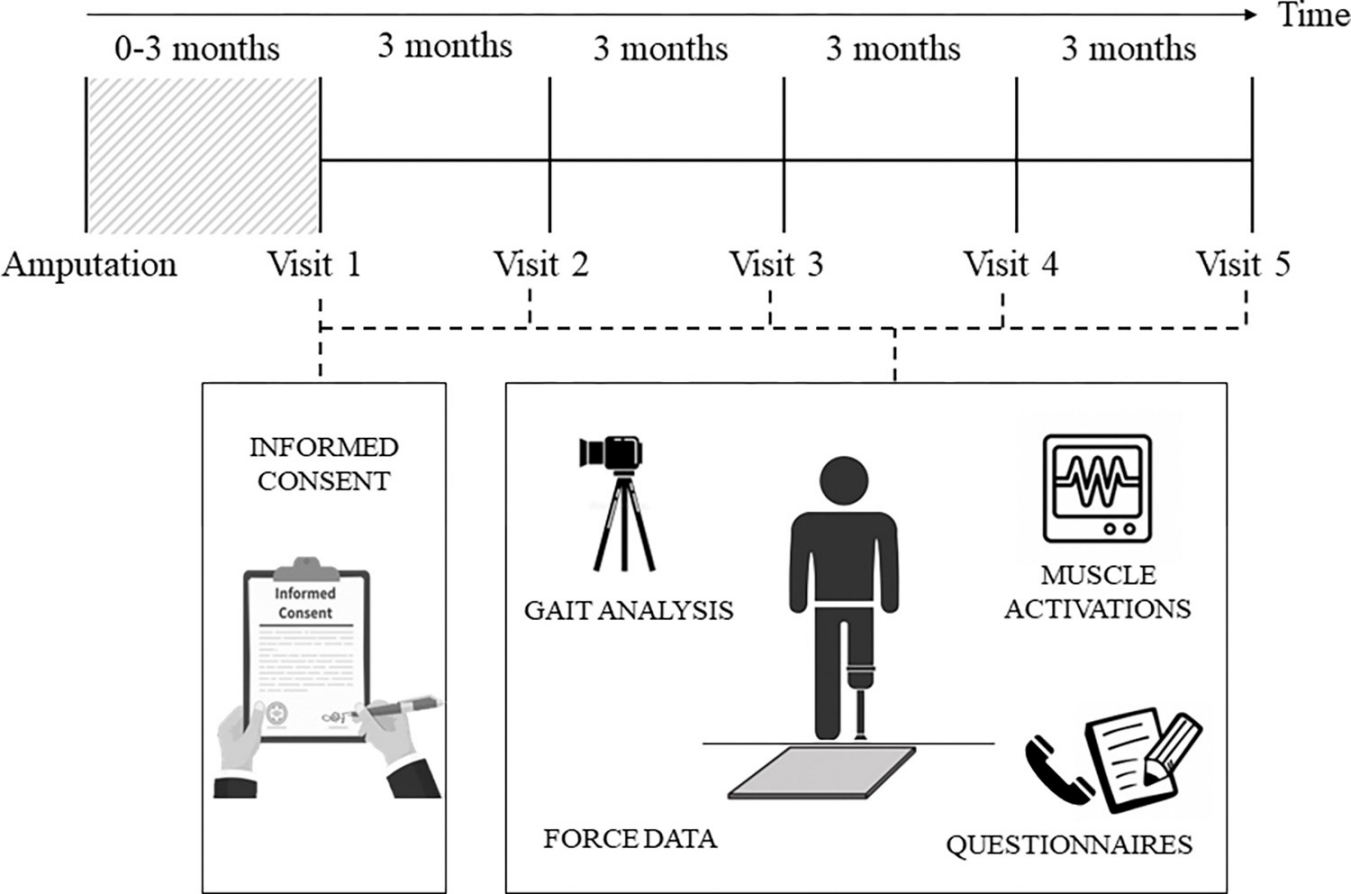
General overview of the study protocol.

This research is a prospective longitudinal study that aims to explore the potential mechanistic pathways of LBP development amongst persons with unilateral LLA. The dataset will include data collection every 3-months over a 12-month period (Fig 1). All testing procedures will take place at the local rehabilitation centre where patients are currently registered for their post-amputation rehabilitation (either the Bristol Centre for Enablement, North Bristol NHS Trust or the Portsmouth Enablement Centre, Portsmouth Hospitals University NHS Trust). Measurement sessions will occur following patients’ routine visits to the centres, ensuring that the testing sessions are structured around the patient’s availability to improve study recruitment and retention. Biomechanical gait variables and muscle activations of trunk and sound lower-limb muscles will be monitored, and objective measurements of different sources of pain and physical activity will be determined using online questionnaires.

### Study population

The recruitment of 30 NHS patients, over 18 years old, with unilateral TFA and TTA will be attempted from the Bristol Centre for Enablement and the Portsmouth Enablement Centre. These centres host a large heterogeneity of patients, allowing us to recruit individuals with different levels of amputation (above/below the knee) and cause of amputation (traumatic/vascular/cancer causes). Furthermore, the rehabilitation programme provided to patients across these centres are very similar regarding aims and outcomes, and the present study will run in parallel with the patients’ personal goals.

### Recruitment

Recruitment started on 01/09/2023 and it will continue for 11-months. The inclusion and exclusion criteria are presented in Table 1. Clinicians at the centres will identify participants who meet the inclusion criteria for study participation during an assessment day (S1 Fig). These clinicians will be existing members of the patients clinical care team and will make the initial approach to patients for study inclusion. Patients will meet the investigator team at the NHS centre where they are currently booked for their rehabilitation in the post-operative period. Participants will be given an information sheet and will sign a consent form as approved by HRA ethics (REC reference: 23/EE/0090, 28/04/23).

**Table 1:** Inclusion/exclusion criteria which must be met by participants before study involvement.

Inclusion	Exclusion
>18 years old	Balance disorders
Unilaterally amputated above or below the knee	Patients who lack the capacity to consent for prosthetic use
Able to walk on level ground	Patients at serious risk of sound foot which limits normal rehabilitation
Newly fitted with a prosthetic (0-3-months post-amputation)	Transfer-in patients
Cause of amputation due to vascular, trauma or cancer	Persons with congenital lower-limb amputation

### Study outcomes

Participants will start the protocol as soon as they are fitted with the prosthesis and they are able to walk (approximately 0-3-months post-amputation). The independent variable of this study is the time course (12-months) with measurements taken every 3-months for 12-months (5 visits in total). The biomechanical and psycho-social variables reported in Table 2 represent the dependent variables. Preliminary pilot tests have been performed in the clinical setting on able-bodied participants.

**Table 2:** Full list of biomechanical and psycho-social variables for analysis.

Variables	Outcome measures
Bio-behavioural	Lower-limb and lumbar kinetics (e.g. joint reaction forces)
	Lower-limb kinematics, including lumbopelvic and lower-limb region movement asymmetries (e.g. joint angles)
	Trunk and lower-limb muscle activations and timings
	Body centre of mass position and symmetry indices
	Temporal-spatial characteristics of gait (e.g. stride length, walking speed)
	Lower-limb-length discrepancies
	Pain intensity/location/type
Psycho-social	Pain attention and interference
	Fear of movement
Psycho-social	Physical activity and quality of life scores
	Anxiety and depression
Other	Anthropometric measurements, socket comfort, prosthesis type, presence of blisters

### Study procedures

Basic anthropometric data (mass, height) will be recorded for each participant before starting the motion capture sessions. Participants will wear their own passive prosthesis, and will perform a static, standing calibration trial in the anatomical position followed by twelve 10-metre gait trials at a comfortable self-selected velocity and six sit-to-stand movements from a chair. A good walking trial will require participants to walk at constant velocity with the foot within the boundary of the force plate. During the sit-to-stand tests, participants must have their feet placed shoulder width apart on the force plate and rise from the chair without the use of the hands/arms.

### Walking trials

**Fig 2:**
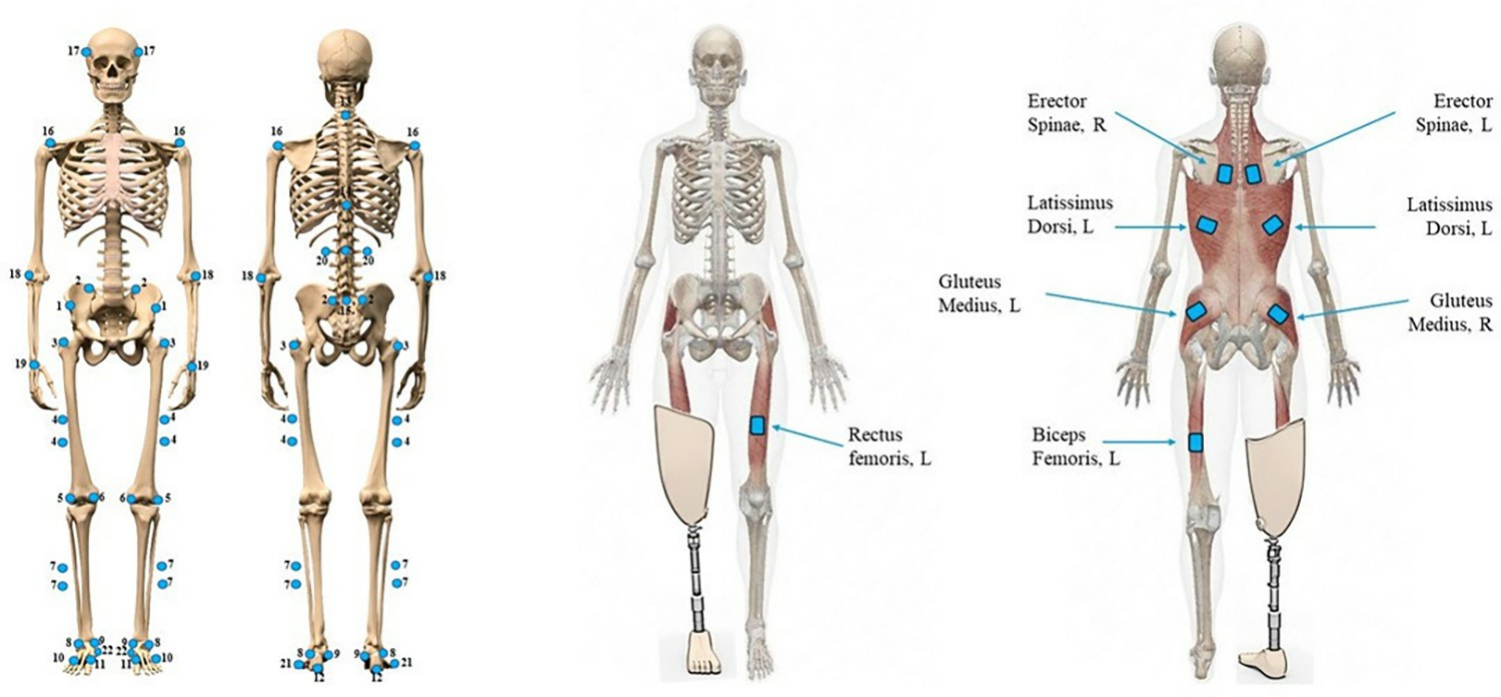
Full body marker set (left) and EMG sensors locations (right). See S1 Table for a completed list of the names of the markers. The rectus femoris and biceps femoris EMG sensors are located on the sound limb, which in this specific example is the left side. However, they are located accordingly with the side of the amputation.

Biomechanical gait analysis will be measured using eight Miqus M5 Qualisys cameras (Qualisys, Sweden), two video cameras, and one portable Kistler force plate (Kistler, Switzerland) to examine movement patterns and loads acting on the joints during level walking over a distance of 10-metres. Each participant will complete twelve walking trials per session (6 trials hitting the force plate with prosthetic limb and 6 trials with the sound limb). Non-invasive reflective markers (n = 54; Fig 2) will be placed on the participants before starting data collection in the anatomical locations listed in S1 Table, and in line with the methods by Silverman and colleagues [56]. Reliability for marker placement in a similar demographic population in terms of BMI (age-matched BMI above the 97th percentile) has been assessed by Horsak and colleagues [57]. The standard error of measurement (SEM) was below 5° for all participants (n = 11) for 28 kinematic parameters measured, including anterior pelvic tilt, pelvis rotation, and hip and knee flexion. Furthermore, 23 parameters displayed acceptable ICCs (≥0.70), and the remainder showed moderate values.

Eight Delsys Trigno EMG electrodes (Delsys, MA, USA) will be positioned bilaterally on four different pairs of muscles (as displayed in Fig 2), to measure their activity. The skin will be adequately prepared by shaving and cleaning with alcohol wipes and the correct location of marker placement will be determined from the SENIAM guidelines [58]. Due to participant characteristics (poor balance and limited mobility) and time constraints (only one hour is allowed for data collection with the patients), the peak activation of each muscle during the sit-to-stand trials will be used for the EMG normalisation analysis, as other authors did in similar investigations on participants with LLA [59, 60]. Variability of EMG placement of lower-limb muscles has been assessed amongst persons with LLA by Wentink and colleagues [61]. The overall inter-subject variability was larger in persons with LLA compared to controls, with a 3% SEM of the EMG data for the stance and swing phases for controls, and 4% for persons with LLA (outliers ranging from 12–14%).

### Sit-to-stand trials

The portable Kistler force plate and the EMG system will be used to determine GRF and muscle mechanics non-invasively to identify potential risks for LBP. Six repetitions will be performed after the walking trials, with the participants still wearing the markers and EMG sensors as described before. During the sit-to-stand tests, participants must have their feet placed shoulder width apart on the force plate, must rise from the chair without the use of the hands/arms, and then hold the standing position which will mark the end of one repetition. Then following variables will be calculated; GRF, centre of pressure (coordinates), trunk kinematics (trunk angles and angular velocity), EMG muscles activity, and timing of the sit-to-stand movement (using the position of the T12 marker and hip/knee peak extension).

### Questionnaires

Participants will complete eight questionnaires, assessing pain and psychological variables (see S2 Table), using the online software Qualtrics. Patients will complete the questionnaires online within 24-hours of each testing session and the principal investigator will provide support via a phone call. Different types of LBP, including both acute and chronic LBP, will be captured from the questionnaires in order to analyse the full spectrum. Additionally, a box for free comments will be included in the questionnaires (see S2 Fig).

These measures have been validated for use amongst the chronic non-malignant pain population and are widely used in clinical settings for the treatment of chronic pain [62]. The Brief Pain Inventory (α > .70), Pain Catastrophising Scale (α = .87), and the TSK (α = .95) are valid and reliable for use with LBP patients [63–65], however reliability has not been assessed amongst persons with LLA.

### Sample size

The sample size of 30 participants was calculated using G*Power software (3.1.9.7). An *a priori* power analysis was performed based on data from a previous study examining lumbar spine kinematics and LBP amongst persons with unilateral LLA [17]. The estimated sample size was calculated to be N = 26 participants (α = 0.05, 1-b = 0.80), with an effect size of 1.03. However, due to possible challenges with recruiting and retaining participants from clinical practice environments, such as people with limb loss, it is important to account for a 10% drop-out rate [66]. Therefore, the recruitment of at least 30 participants in total will be attempted from the NHS Enablement Centres.

It is important to note that the sample size calculation takes into account only the kinematic variables, and not the relationship with the psycho-social variables measured through the online questionnaires. Ideally, a much larger population would be required for powered analysis of the psycho-social variables, however, due to the time constraints and the lack of data, the estimated sample size of N = 30 patients is the most feasible for the project duration.

### Data analysis

Marker trajectories and GRF data during the walking and sit-to-stand trials will be processed using a 4^th^-order Butterworth filter with a cut-off frequency of 6 Hz. One complete gait cycle per walking trial will be analysed. Joint kinematic and kinetic data will be time-normalised to 101 sample points per gait cycle. The start of the gait cycle is defined as the instant when the foot hits the platform and the end of the gait cycle is defined by the method proposed by De Asha and colleagues [67]. Joint range of motion will be calculated as the difference between the maximum and minimum angles during a gait cycle. Initial heel-strike and toe-off will be detected from the force trace as a single force platform will be used for all trials. Initial heel-strike will be detected as the first instance of force production, and toe-off will be defined as the final data point of force production. End heel-strike of a single gait cycle will be defined by the time-point of peak hip extension of the contralateral limb [67]. Raw EMG signals will be band-pass filtered between 30 and 400 Hz, full-wave rectified, and low-pass filtered (6 Hz) to create a linear envelope [59, 68]. The peak EMG signals of the trunk and sound lower-limb muscles during sit-to-stand trials will be used to normalise EMG data from the walking and sit-to-stand trials for each individual.

**Fig 3:**
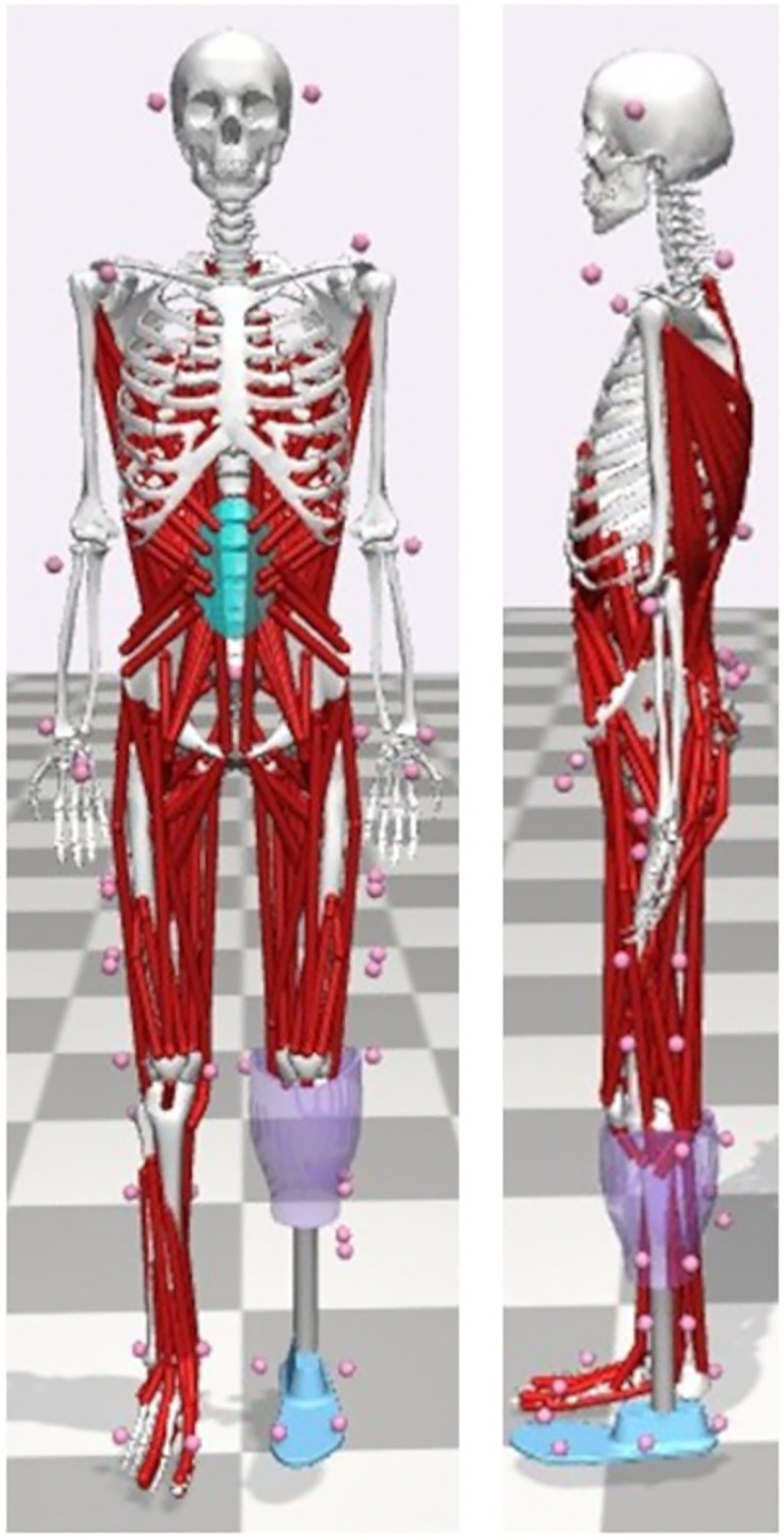
Example of the OpenSim musculoskeletal model for a TTA. The pink balls represent marker locations, and the red strings represent the muscles included in the model.

A 3D full-body TTA and TFA musculoskeletal model (38 degrees of freedom), modified from the validated model described by Rajagopal and colleagues [69], will be used for muscle-driven simulations of gait (Fig 3). The musculoskeletal models were developed in OpenSim 4.3 and includes a total of 324 musculotendon actuators. To model the trunk and pelvis, markers will be placed over the T12 and C7 spinous processes, the first sacral vertebrae, and bilaterally over the anterior superior iliac spine and posterior superior iliac spine. Additionally, a marker cluster will be placed on the lumbar spine region between the T12 and S1 markers. The models will be scaled in size and body mass to each participant using a static, standing calibration trial. The prosthetic limb will be scaled by positioning a marker at the bottom of the socket where it meets the pylon, allowing the pylon and the residual limb to be scaled with different scaling factors [70]. Data will be processed in OpenSim for the calculation of joint kinematics (inverse kinematics) and kinetics (inverse dynamics and joint reaction analysis), using information from the muscles activation (EMG-informed analyses; [71]). A full list of the variables measured for analysis was presented previously in Table 2.

### Statistical analysis

All biomechanical data will be processed in MATLAB® R2023a (MathWorks, Inc) and OpenSim and reported as mean ± standard deviation. Statistical analysis will be conducted using IBM SPSS software (Version 26.0. Armonk, NY). Descriptive data obtained from anthropometrics, online questionnaires, and gait parameters will be analysed by descriptive statistical methods. Biomechanical and psycho-social data will be inputted into a random forest algorithm, which will be used as a dimensionality reduction approach to better explain the variance of the LBP data distribution [72]. Thus, this approach will allow us to evaluate how important each factor is influencing the final outcome (i.e. LBP presence). The process of feature selection is a type of exploratory analysis based on the Pearson’s *r* correlation coefficient to estimate correlations between all the variables collected and LBP. The application of random forest analysis in clinical research is considered a key step towards improving patient related health outcomes [73]. Clinical studies in the literature support the use of random forest analysis for classification performance and prediction, for example in the diagnosis of breast cancer using mammography imaging techniques [74]. Generalised linear mixed models will be used to examine the contributions from biomechanical and psycho-social variables to predict the development of LBP. The variables included in the analysis will be selected based on previous studies [17, 26, 47] and the results from the random forest analysis. Generalised linear mixed models will then be created to identify the best predictors to answer research questions two, three, and four, with pain as a ‘yes/no’ binary variable, and a linear mixed effects model will investigate research question one. Significance will be accepted at *p* < 0.05.

### Research question one

Gait analysis outcome variables and muscle activations will be modelled as the dependent variables, ‘timepoint’ will be the fixed effect of interest, and participants will be defined as a random effect [17].

### Research question two

Pain will be modelled as the dependent variable, ‘timepoint’ will be the fixed effect of interest, and participants will be defined as a random effect.

#### Research question three

Function will be modelled as the dependent variable, ‘timepoint’ and pain will be the fixed effects of interest, and participants will be defined as a random effect.

#### Research question four

Pain and function will be modelled as the dependent variable, ‘timepoint’, biomechanical, and psycho-social variables will be the fixed effects of interest, and participants will be defined as a random effect.

#### Data storage and retention

The Principal Investigator and the academic supervisor will have access to the final data set. All results will be stored (raw and anonymised) on the University of Bath data servers. Participants will provide informed consent at each visit to a testing session before participating. Data will be coded anonymously and accessed only by the research investigators. Data will be stored on a password protected PC used by the Principal Investigator for the data collection and moved onto the University of Bath servers in the next 3 hours following data collection. All data records will be anonymised. Participants who wish to receive their individual results via email will provide personal information.

All hard copies of personal data, such as informed consent forms, will be stored in a locked filing cabinet in the Principal Investigators office for 10 years. Access to this office is restricted to the Principal Investigator, the academic supervisor Dr Elena Seminati and Dr Dario Cazzola.

During the study each participant will be assigned a unique project code, which will be used to identify the participant anonymously. Participants will not be identifiable from any resulting data, which may be published in journals or presented at meetings/conferences. Data and patient information will be collected in accordance with the confidentially NHS Code of Practice, and all information will be subject to the conditions of the Data Protection Act 1998.

### Ethics and dissemination

This research has ethical approval from East of England—Cambridge East Research Ethics Committee HRA ethics (REC reference: 23/EE/0090), granted on 28/04/23. Informed written consent is obtained from the participants by the principal investigator who is a trained research personnel. The study results will be submitted for publication in a peer-reviewed clinical biomechanics journal, and disseminated through conference presentations.

#### Patient & public involvement

Patient and public involvement input was obtained via a patient advisory group at the Portsmouth Enablement Centre reviewing the participant information sheet and lay summary in order to obtain feedback and identify any jargon/medical terms which may need revising. This process ensured that the questions and aims/objectives of the present study are aligned with the patient experience and expectations.

## Supporting information

S1 Fig Recruitment process flow chart. (TIF)

S2 Fig Online questionnaires to be completed by participants within 24-hours of each testing session. (ZIP)

S1 Table Marker names and locations. On the amputated limb, the lower-limb markers will be placed taking into account the centre of rotation of the ankle on the prosthesis (and the knee where relevant for persons with transfemoral amputation), and the intact leg positions. ASIS, anterior superior iliac spine; PSIS, posterior superior iliac spine; MTP5, fifth metatarsal bone; MTP1, first metatarsal bone.
(TIF)

S2 Table Online questionnaires assessing pain and psychological variables. A full version of the questionnaires is available in the supporting information S2 Fig.
(TIF)

S1 File (ZIP)
