# Supplementary figures and images for: Exploring the associations between the biomechanical and psychological mechanistic pathways of lower back pain development amongst persons with lower-limb amputation: A study protocol
Source: PLoS One. 2025 Feb 6;20(2):e0314523. doi: 10.1371/journal.pone.0314523 (PMC11801542; doi:10.1371/journal.pone.0314523)

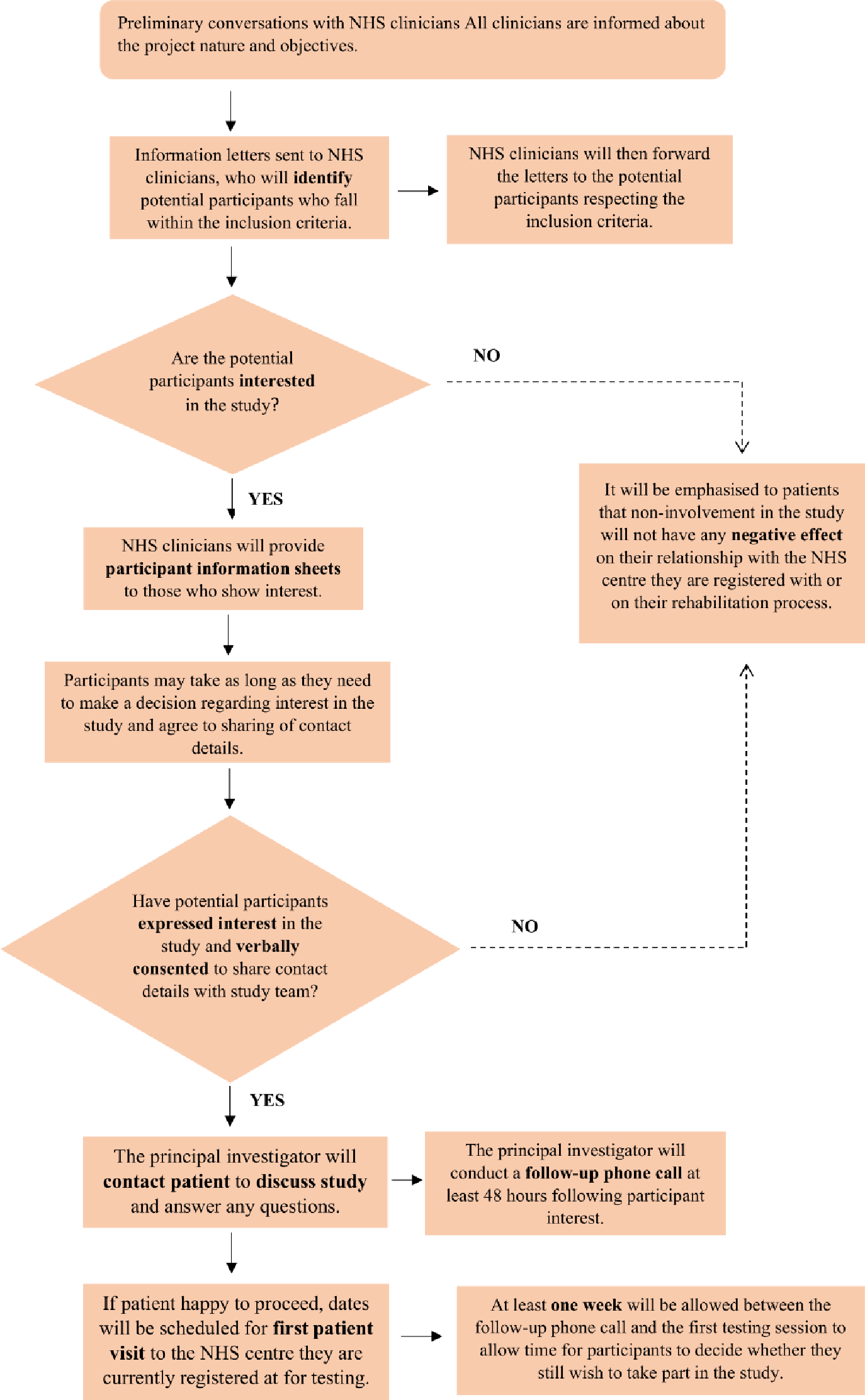

Supplement: S1 Fig — (TIF) [file pone.0314523.s001.tif]

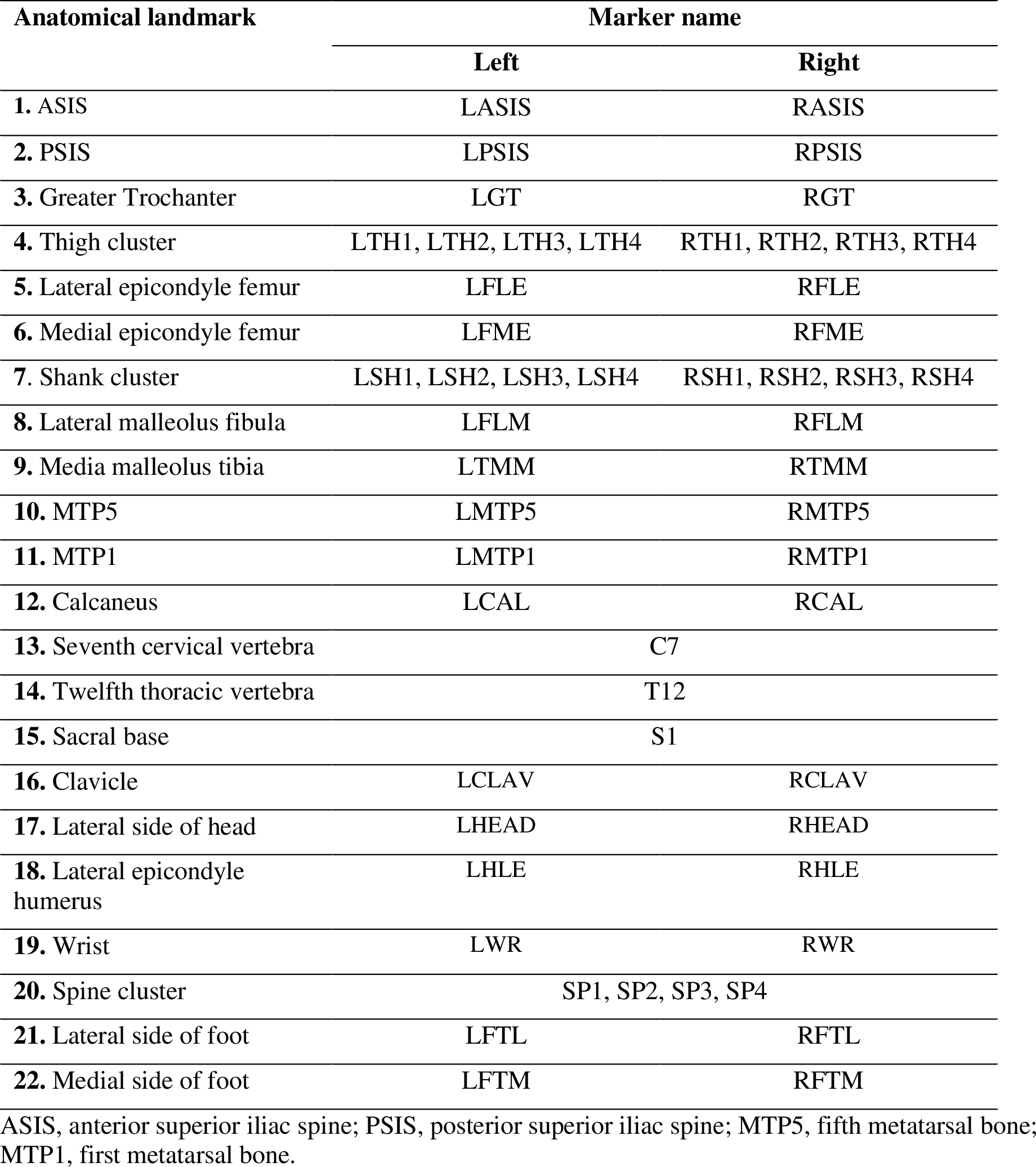

Supplement: S1 Table — On the amputated limb, the lower-limb markers will be placed taking into account the centre of rotation of the ankle on the prosthesis (and the knee where relevant for persons with transfemoral amputation), and the intact leg positions. ASIS, anterior superior iliac spine; PSIS, posterior superior iliac spine; MTP5, fifth metatarsal bone; MTP1, first metatarsal bone. (TIF) [file pone.0314523.s003.tif]

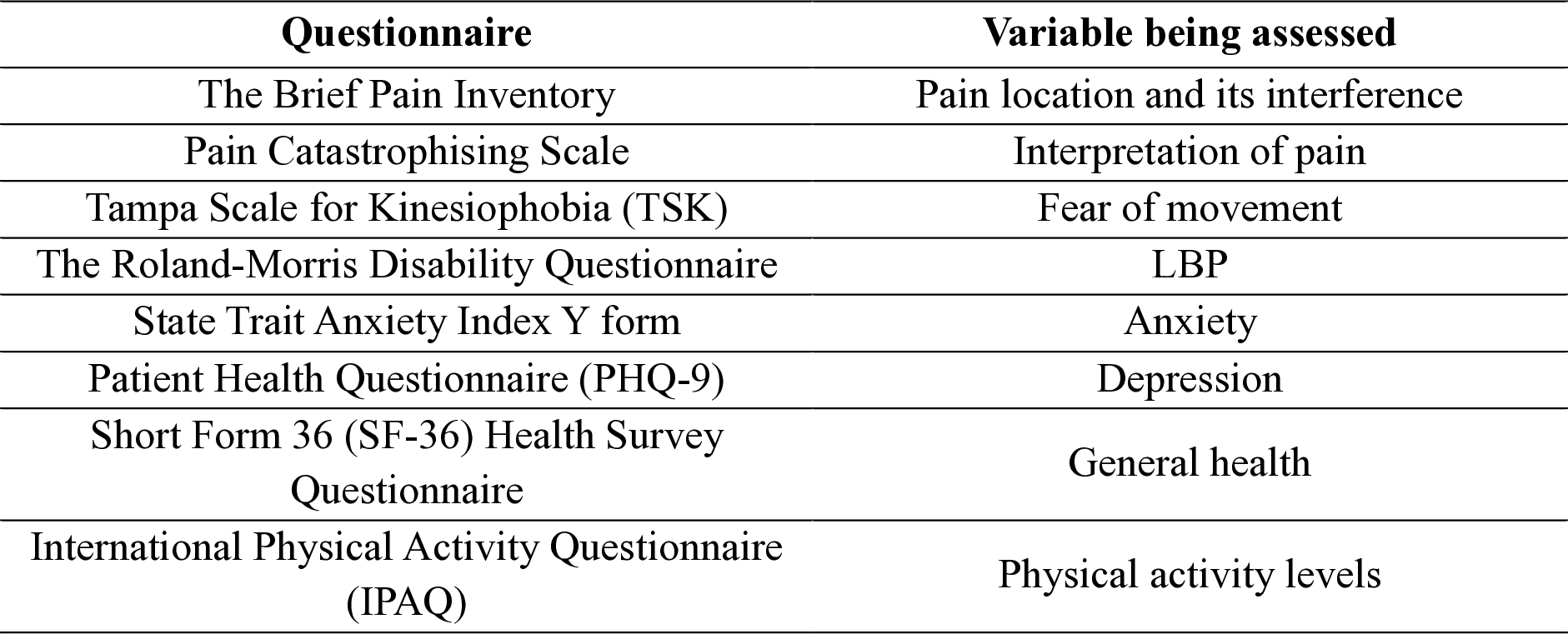

Supplement: S2 Table — A full version of the questionnaires is available in the supporting information S2 Fig. (TIF) [file pone.0314523.s004.tif]

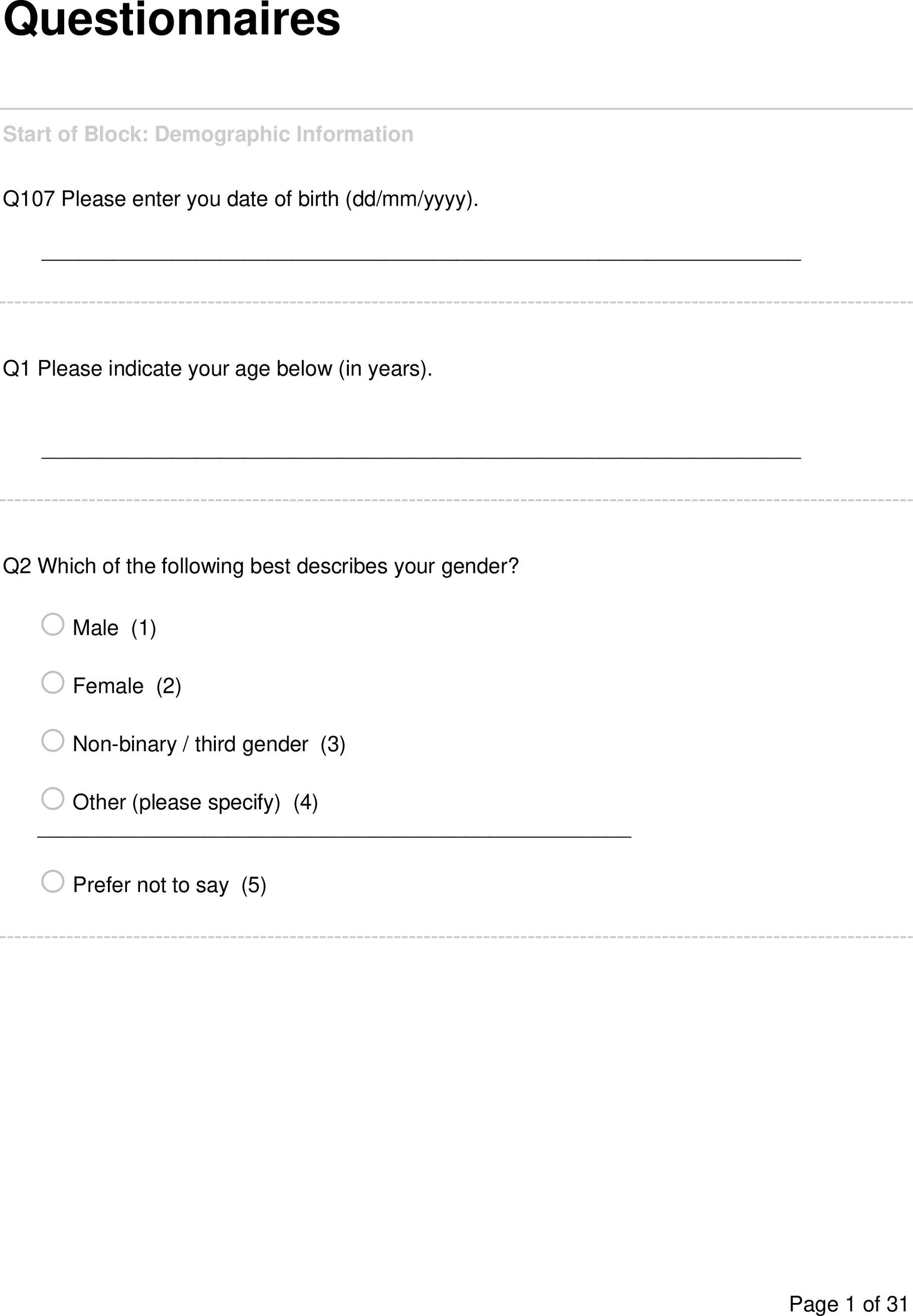

Supplement: S1 File — (ZIP) [file pone.0314523.s005.zip › PACE Corrected/S2_Fig.tif]

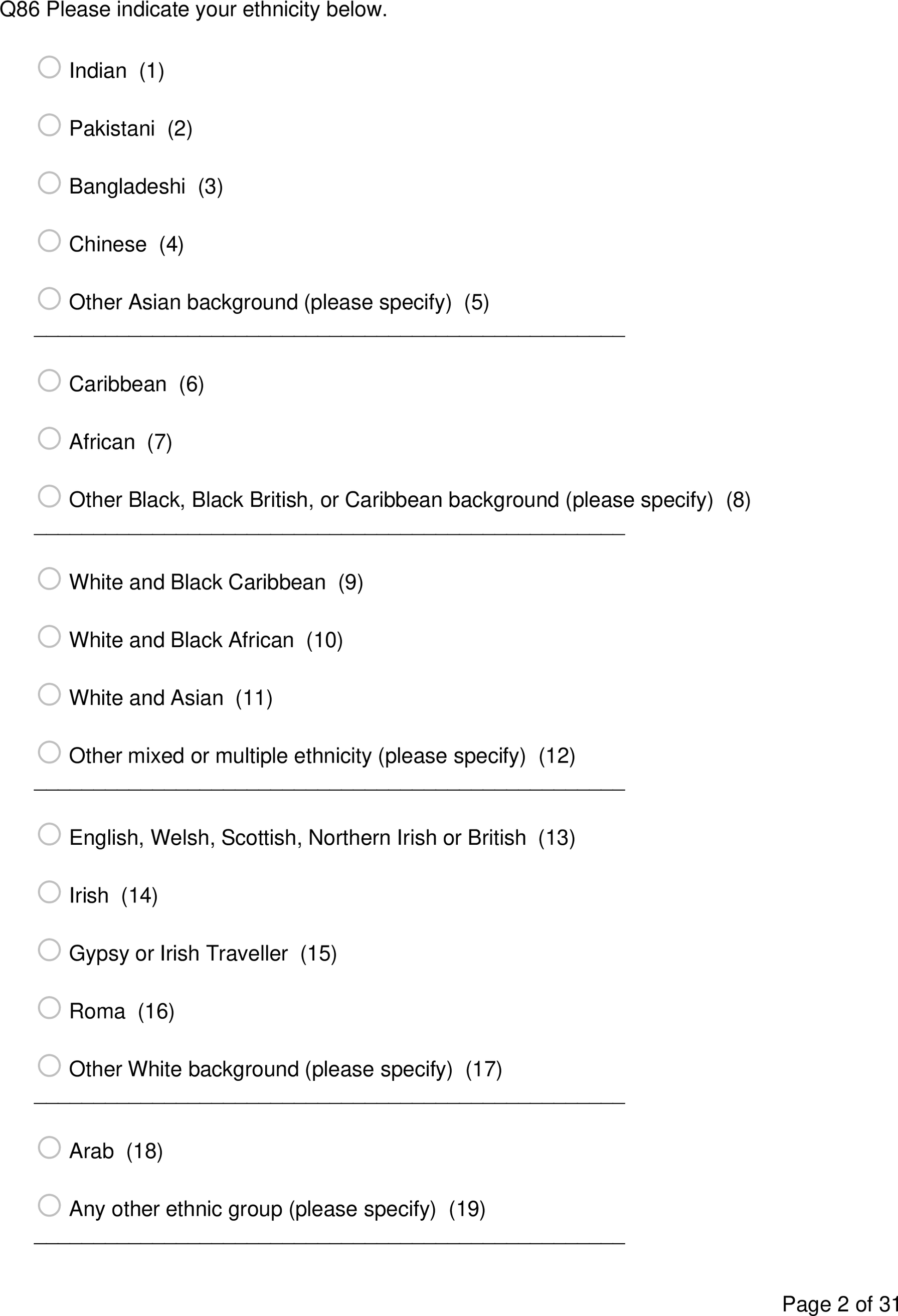

Supplement: S1 File — (ZIP) [file pone.0314523.s005.zip › PACE Corrected/S2_Fig.tif]

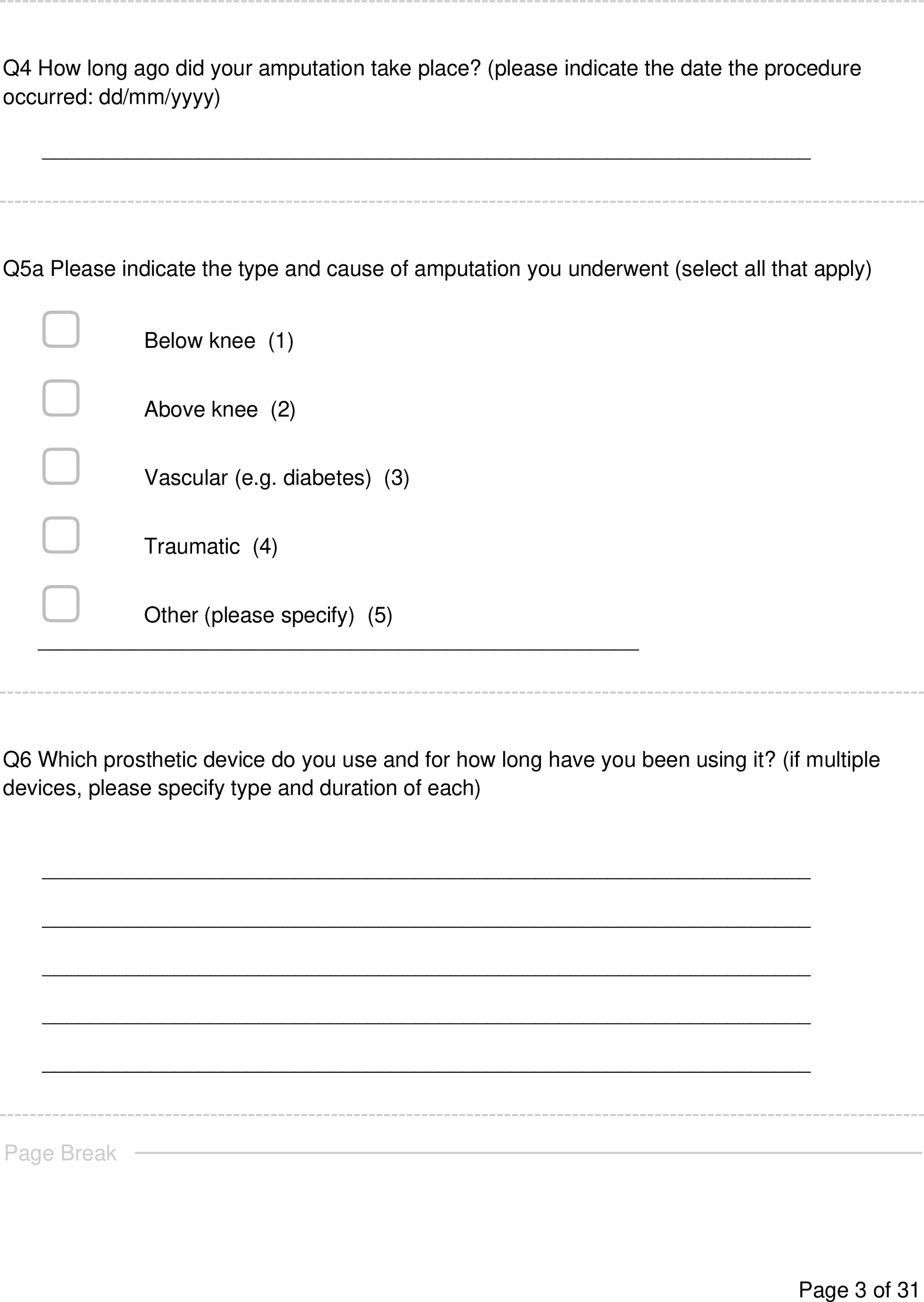

Supplement: S1 File — (ZIP) [file pone.0314523.s005.zip › PACE Corrected/S2_Fig.tif]

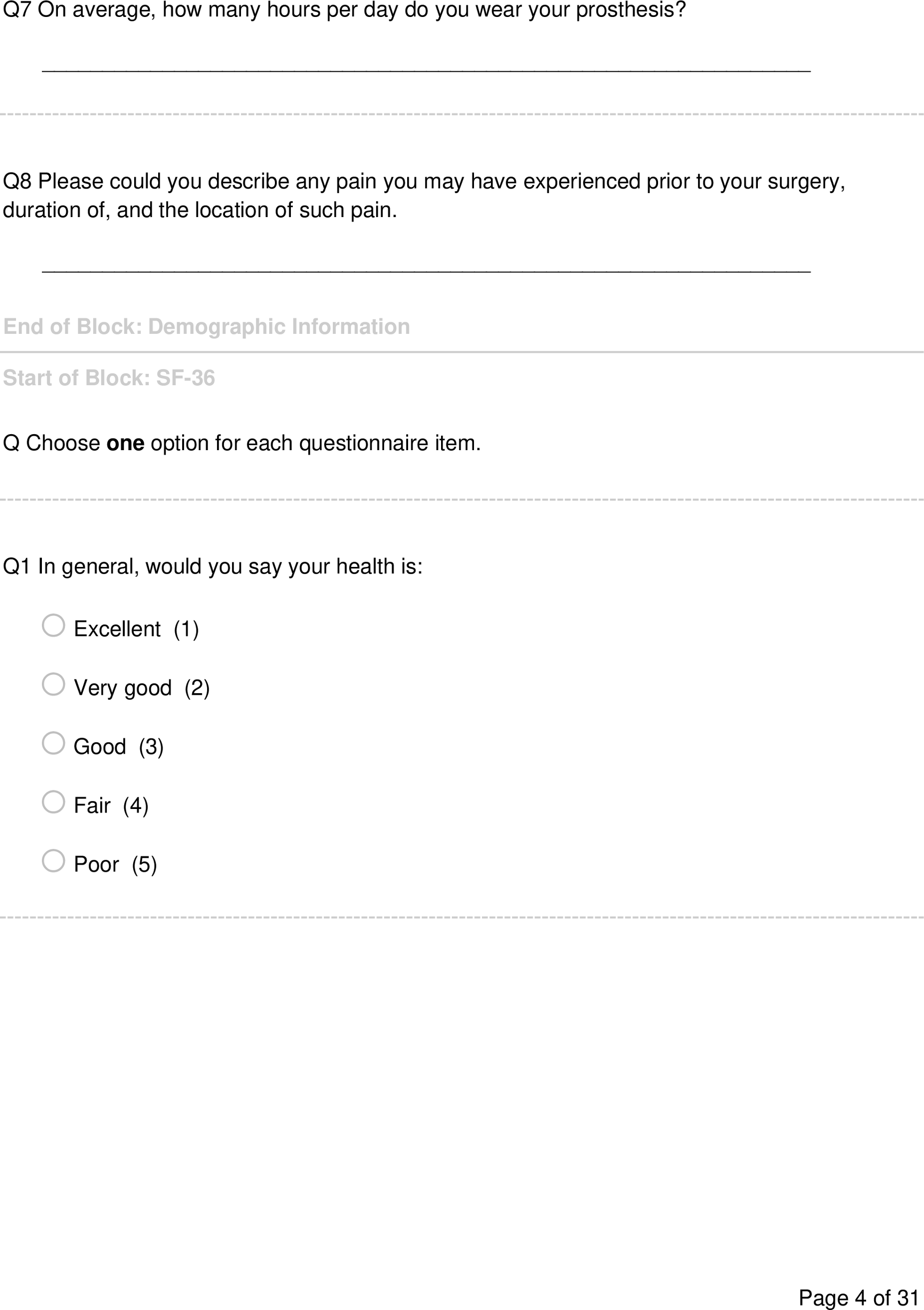

Supplement: S1 File — (ZIP) [file pone.0314523.s005.zip › PACE Corrected/S2_Fig.tif]

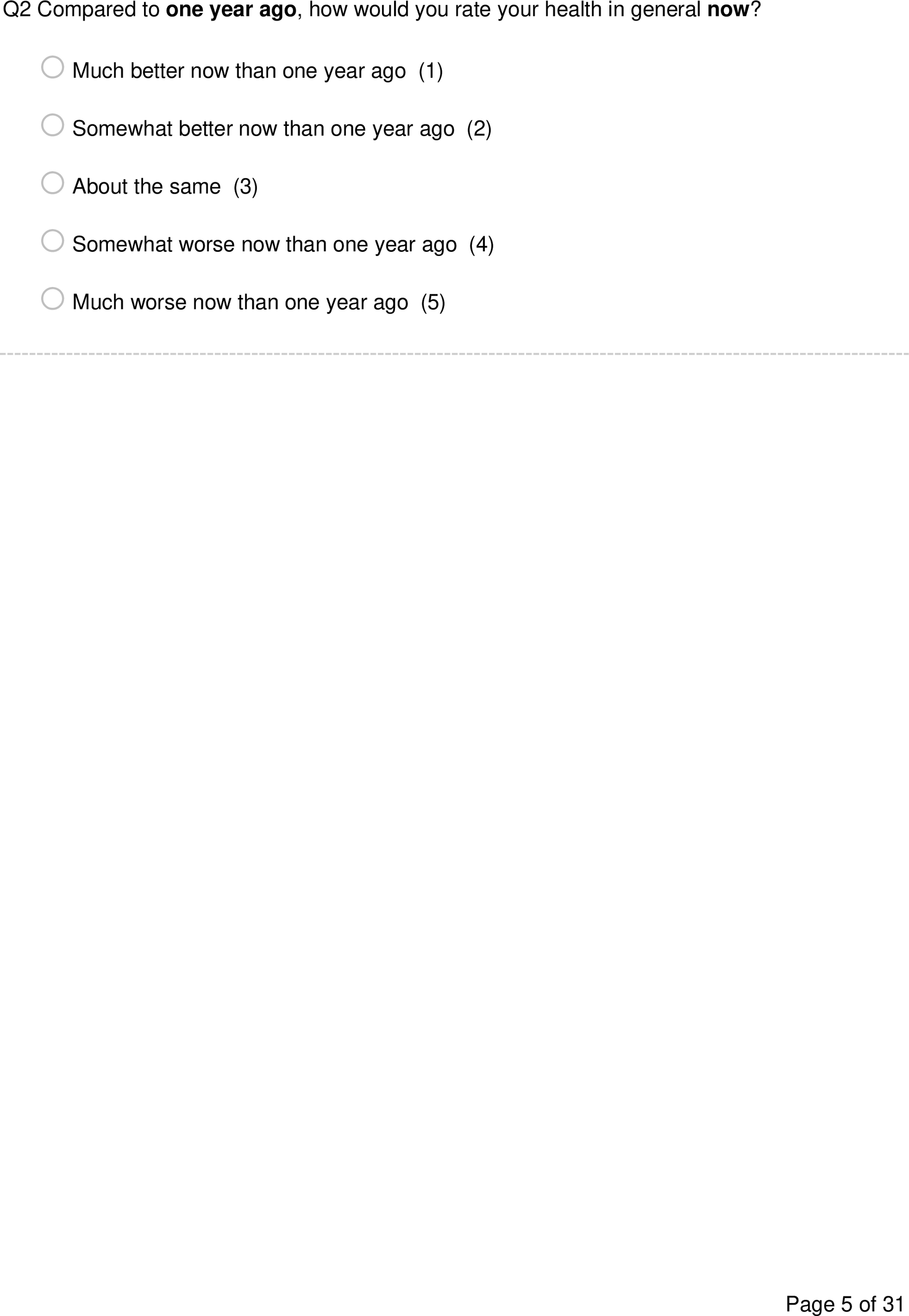

Supplement: S1 File — (ZIP) [file pone.0314523.s005.zip › PACE Corrected/S2_Fig.tif]

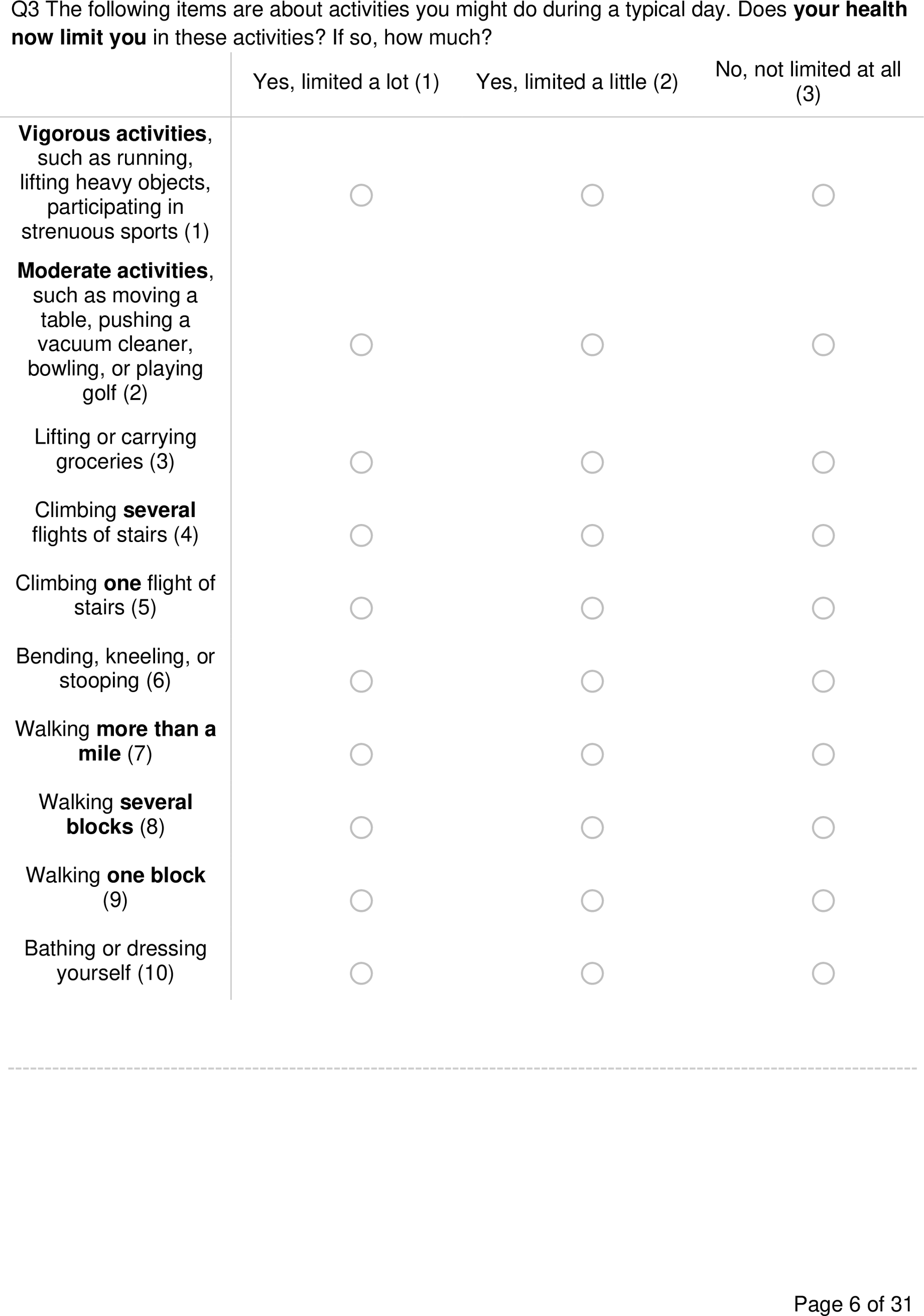

Supplement: S1 File — (ZIP) [file pone.0314523.s005.zip › PACE Corrected/S2_Fig.tif]

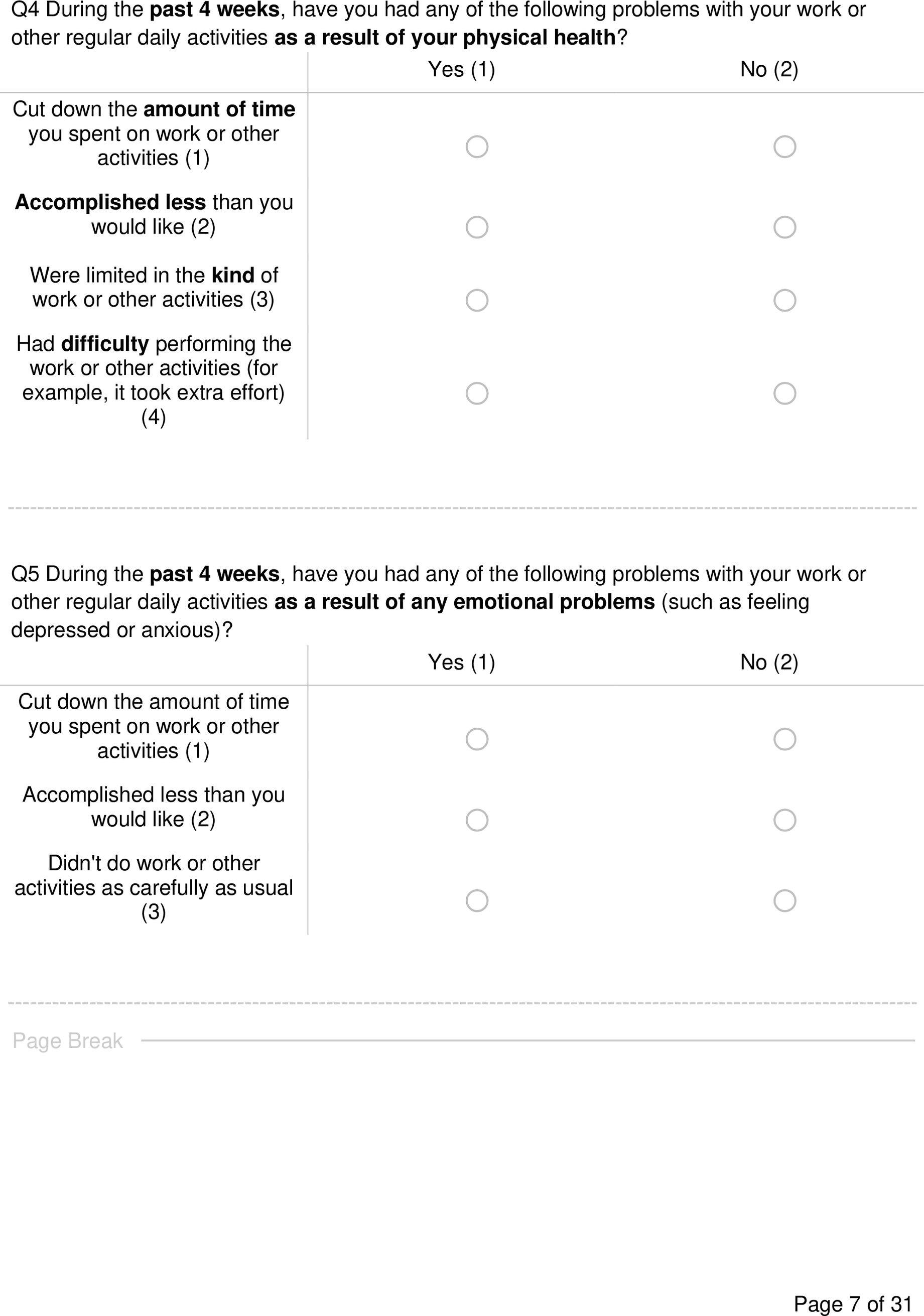

Supplement: S1 File — (ZIP) [file pone.0314523.s005.zip › PACE Corrected/S2_Fig.tif]

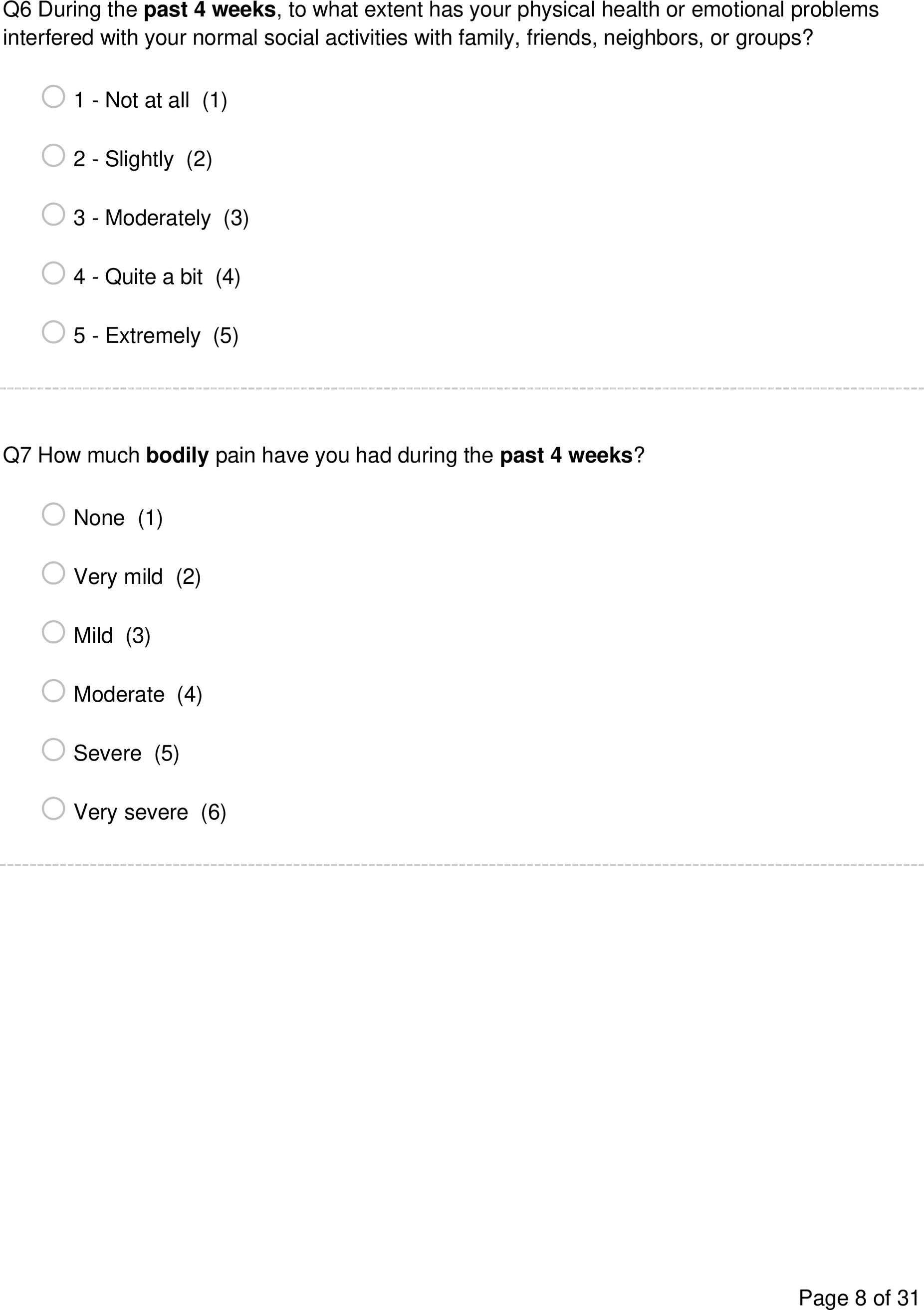

Supplement: S1 File — (ZIP) [file pone.0314523.s005.zip › PACE Corrected/S2_Fig.tif]

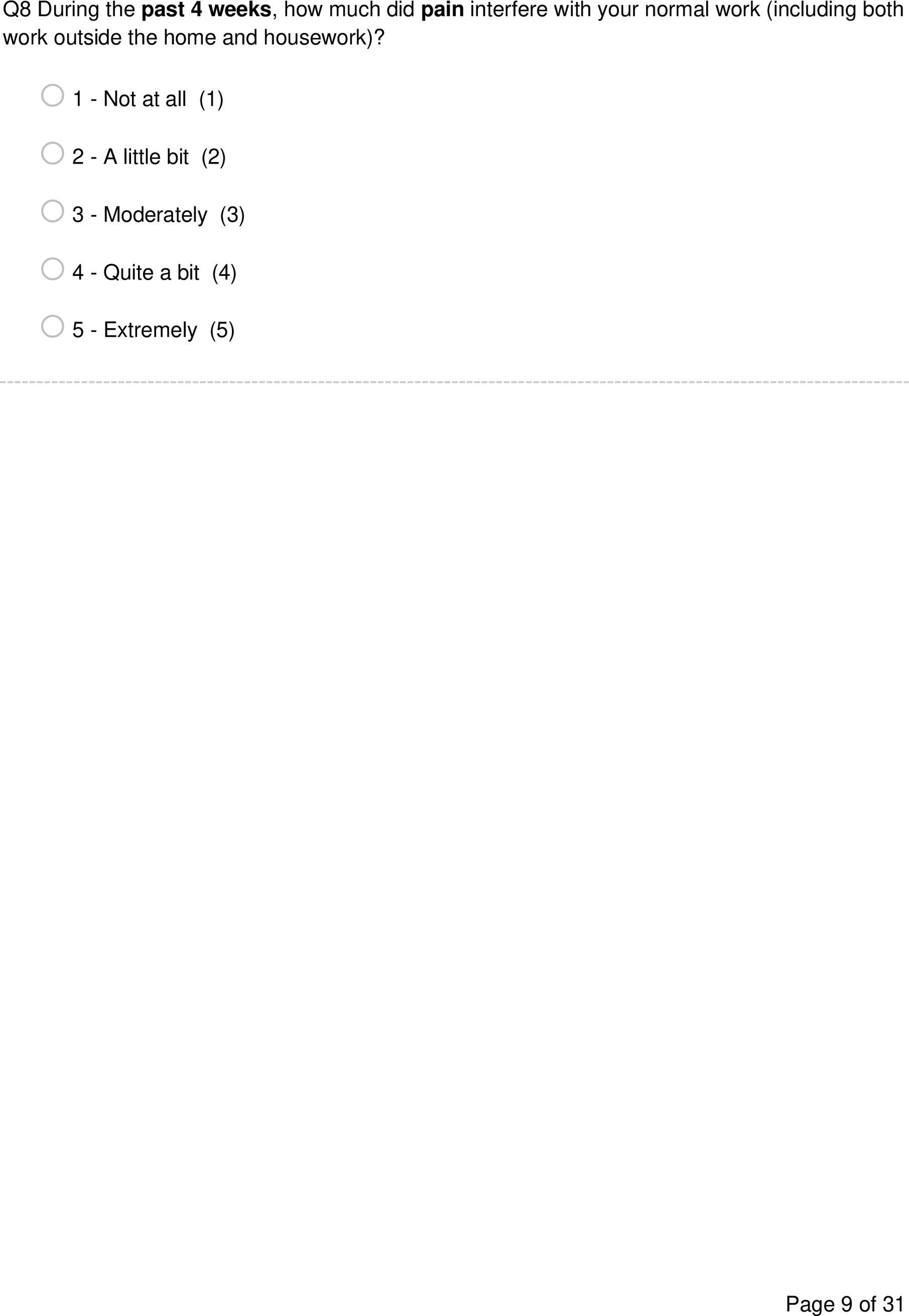

Supplement: S1 File — (ZIP) [file pone.0314523.s005.zip › PACE Corrected/S2_Fig.tif]

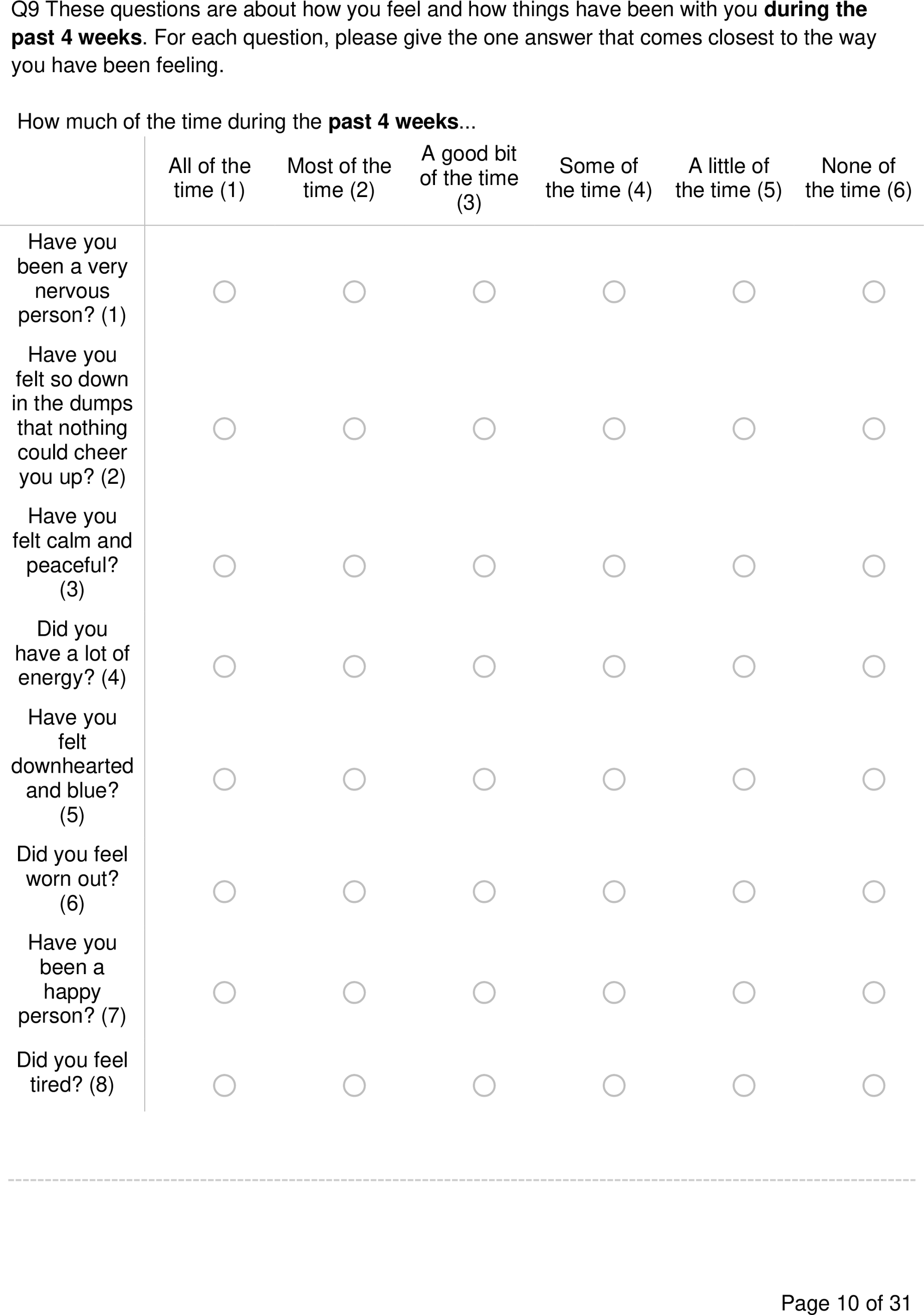

Supplement: S1 File — (ZIP) [file pone.0314523.s005.zip › PACE Corrected/S2_Fig.tif]

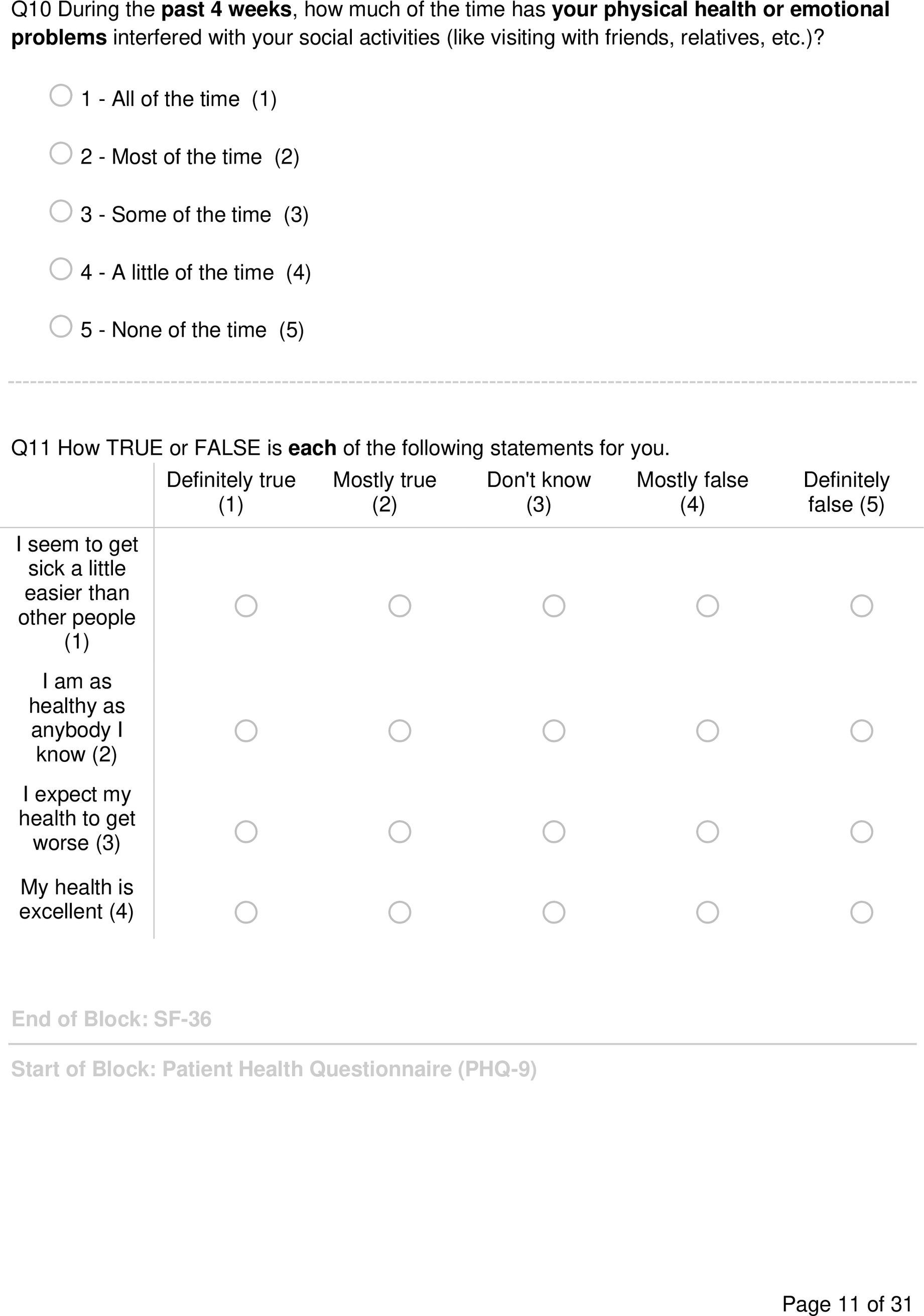

Supplement: S1 File — (ZIP) [file pone.0314523.s005.zip › PACE Corrected/S2_Fig.tif]

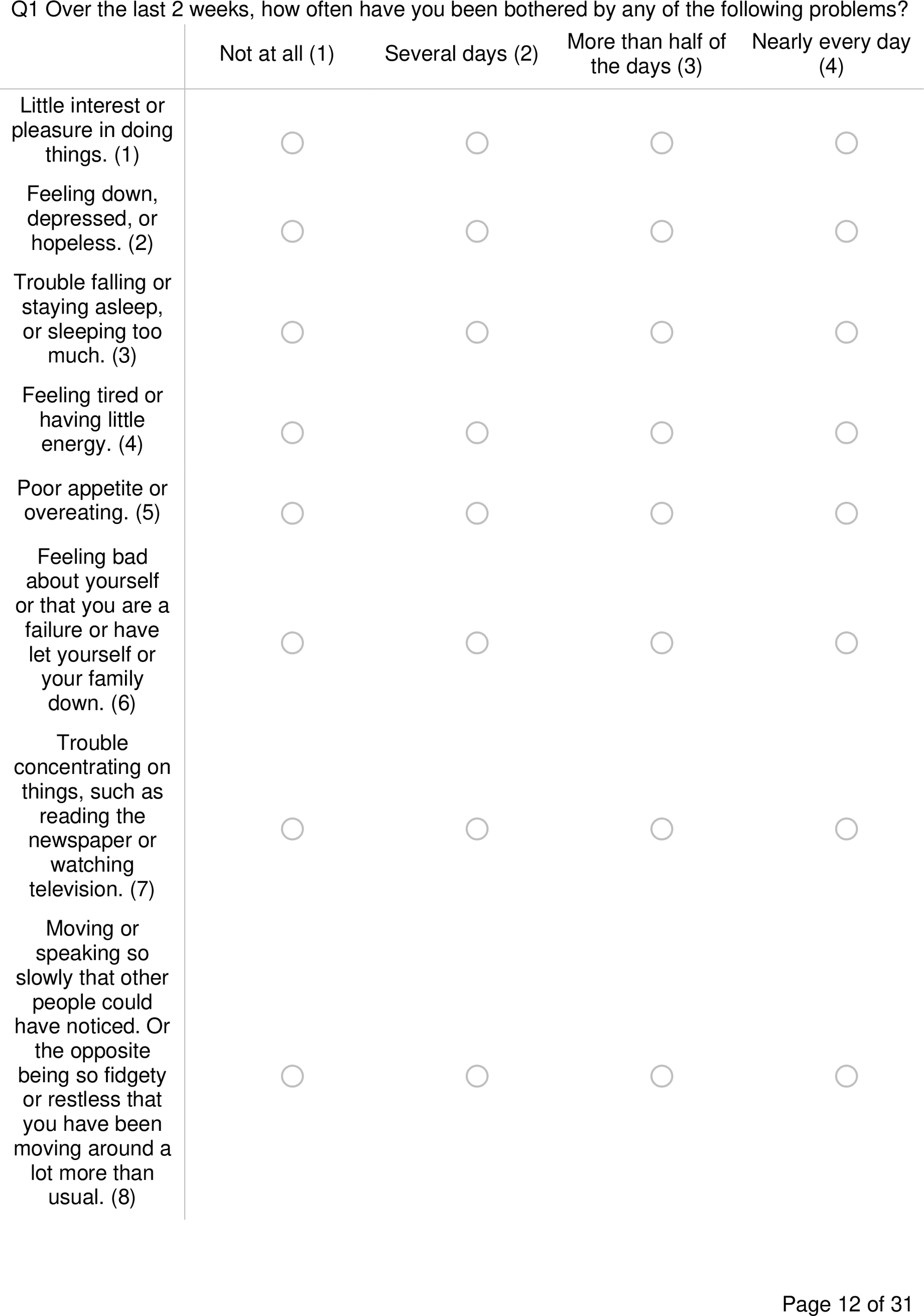

Supplement: S1 File — (ZIP) [file pone.0314523.s005.zip › PACE Corrected/S2_Fig.tif]

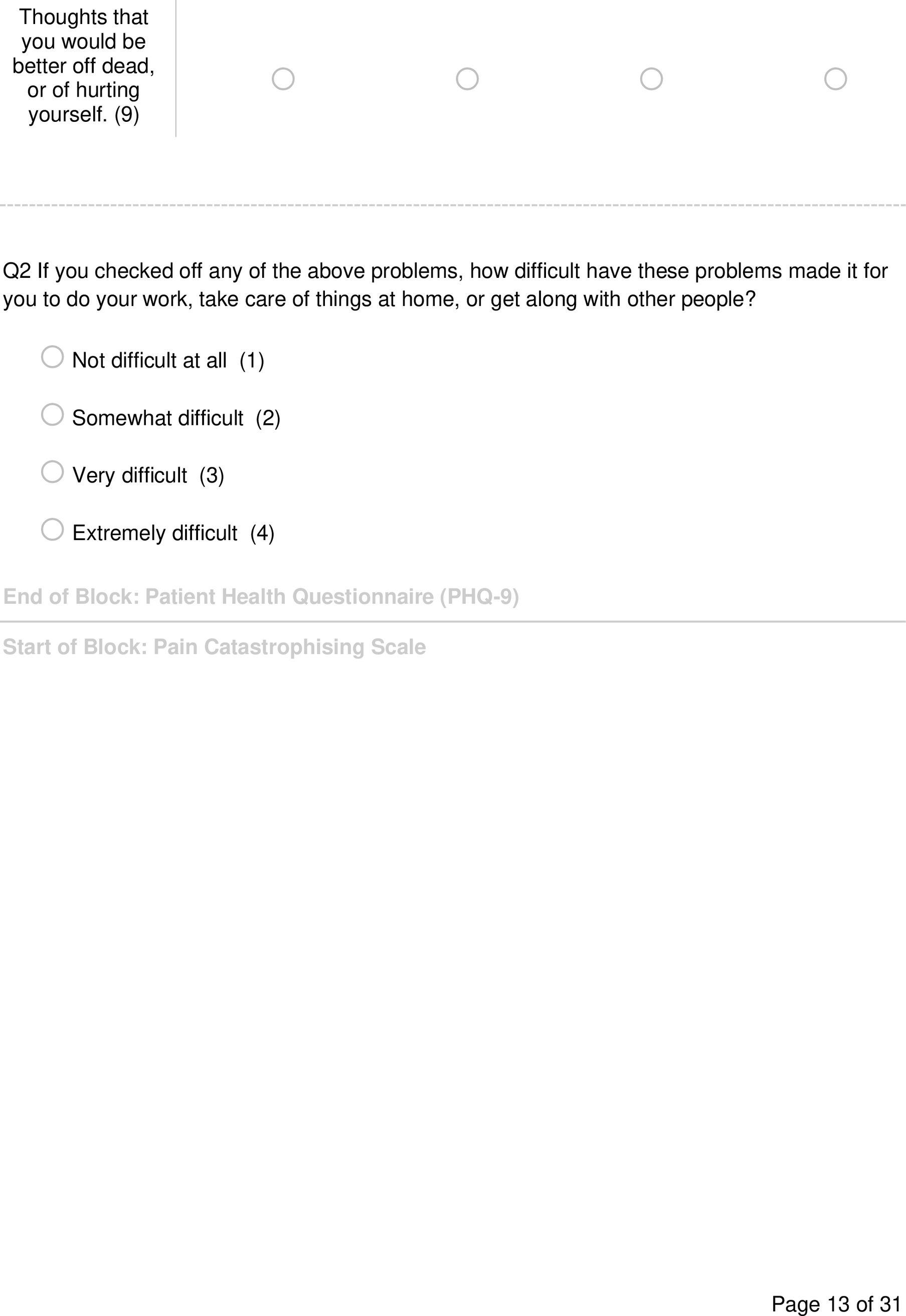

Supplement: S1 File — (ZIP) [file pone.0314523.s005.zip › PACE Corrected/S2_Fig.tif]

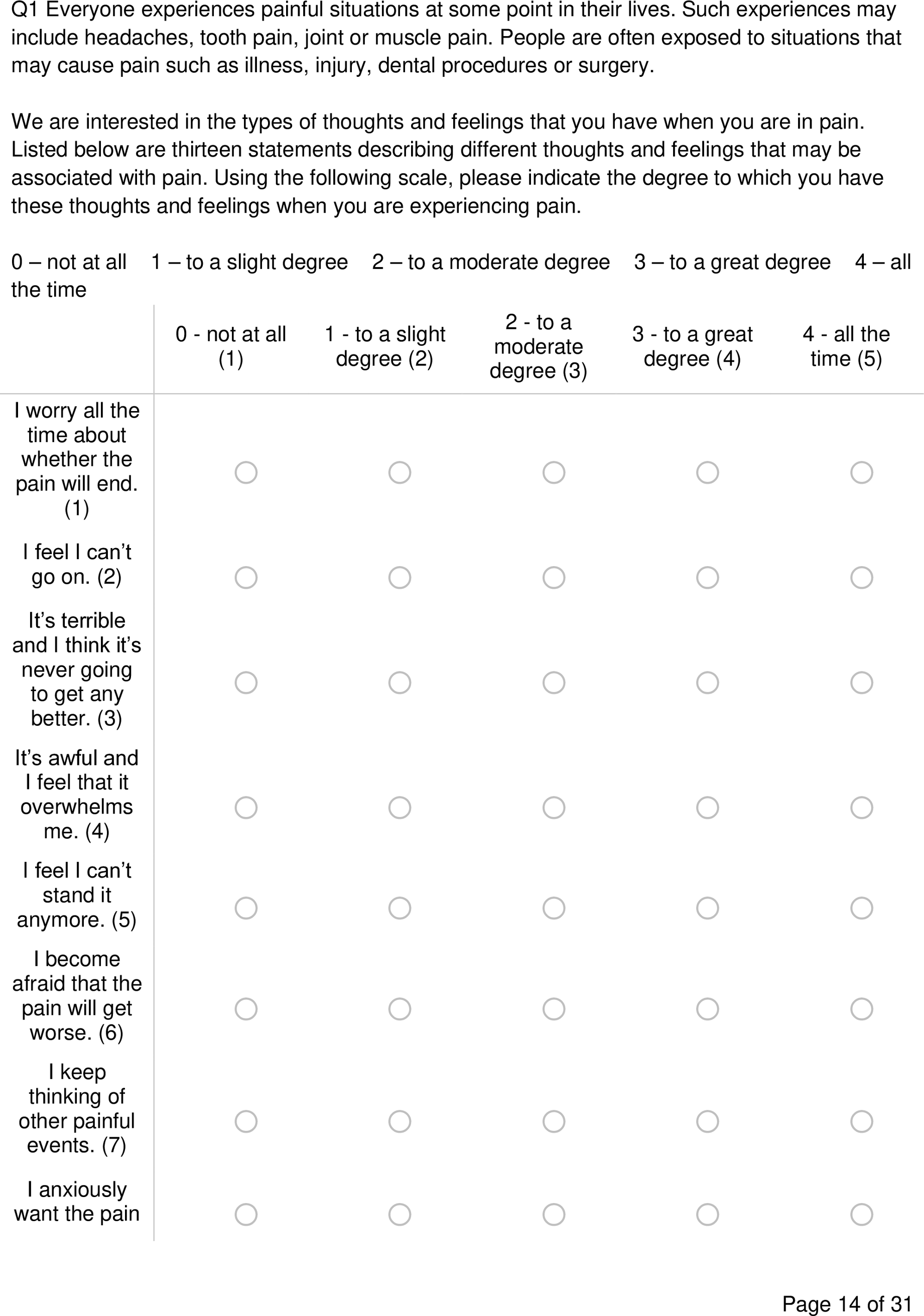

Supplement: S1 File — (ZIP) [file pone.0314523.s005.zip › PACE Corrected/S2_Fig.tif]

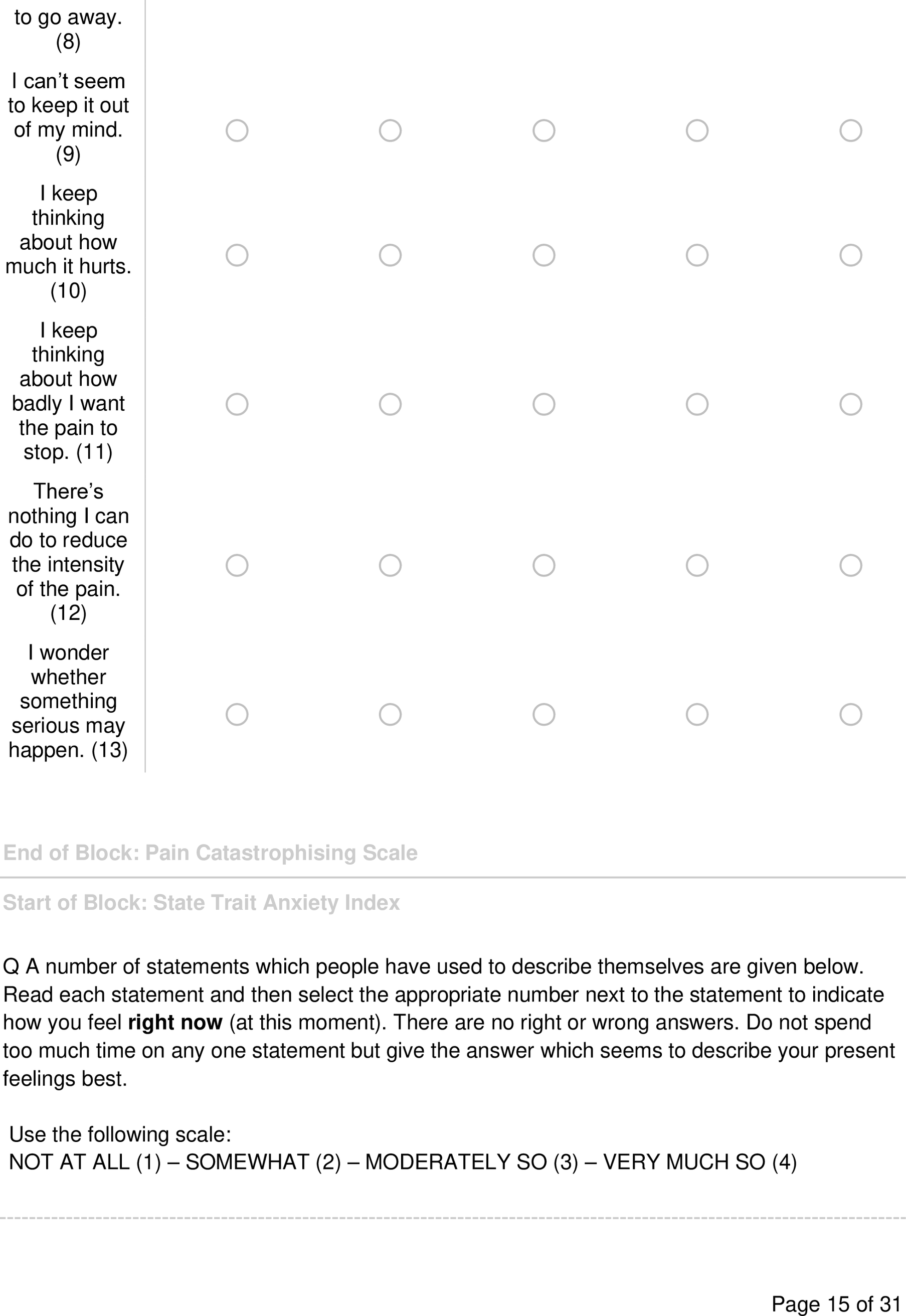

Supplement: S1 File — (ZIP) [file pone.0314523.s005.zip › PACE Corrected/S2_Fig.tif]

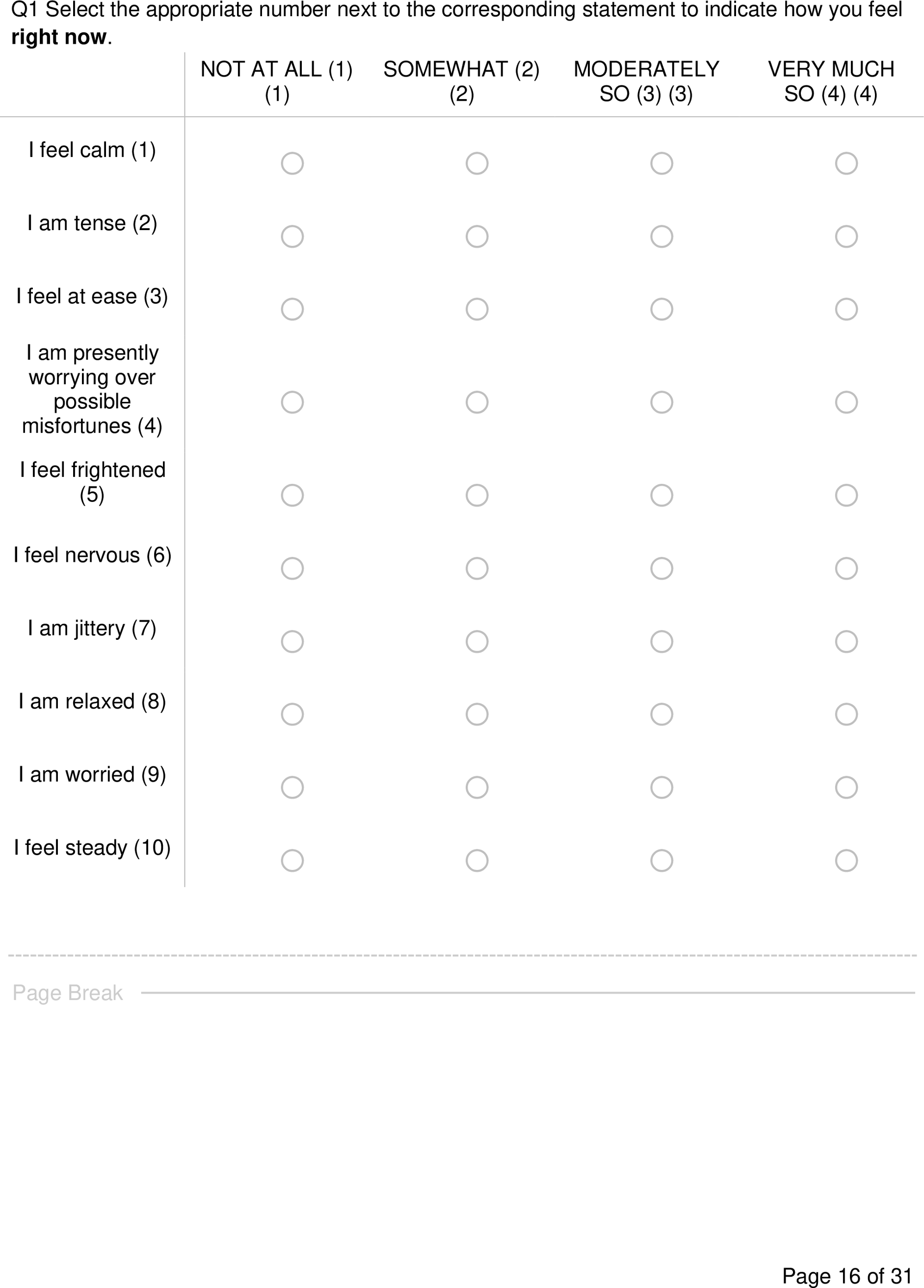

Supplement: S1 File — (ZIP) [file pone.0314523.s005.zip › PACE Corrected/S2_Fig.tif]

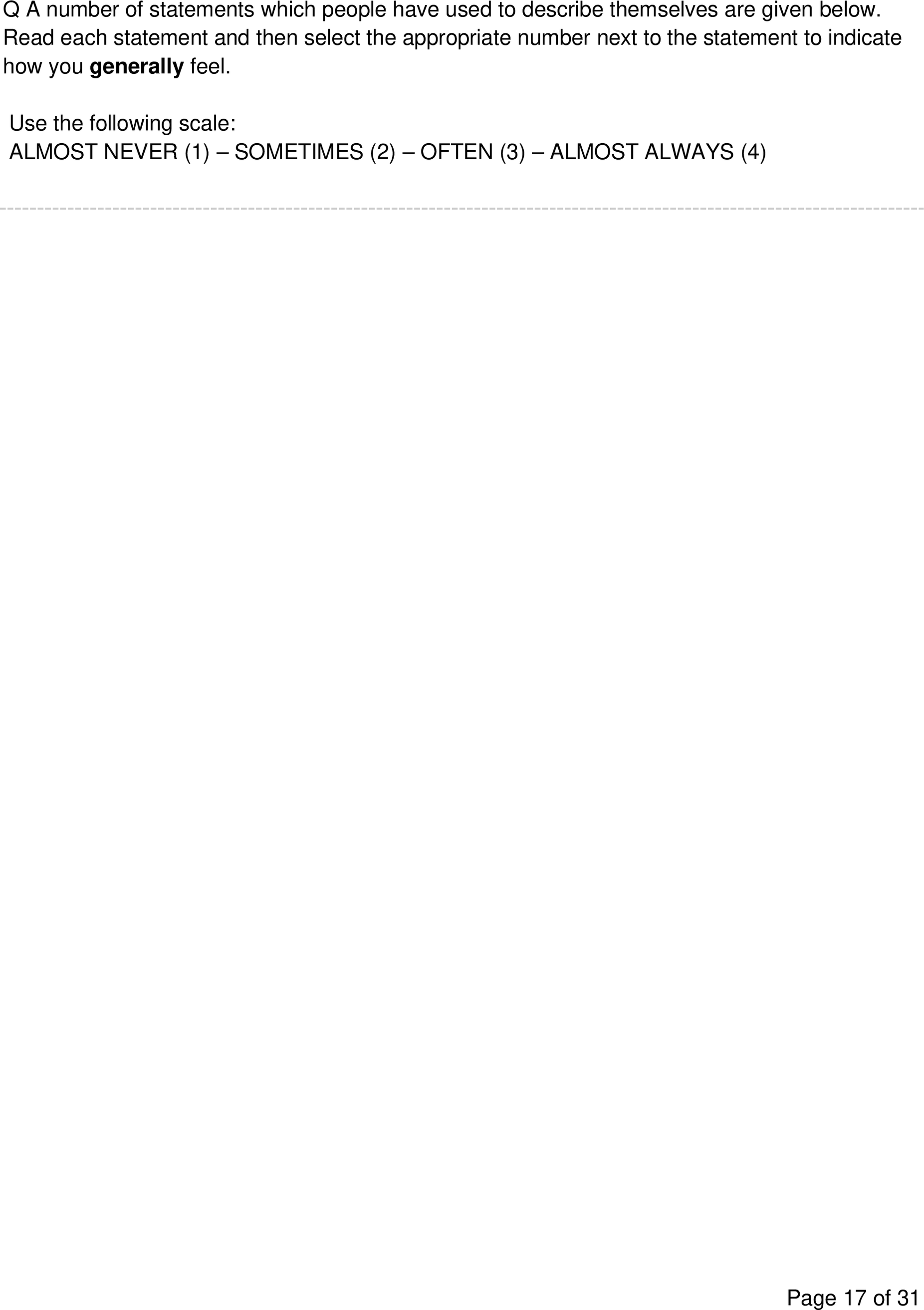

Supplement: S1 File — (ZIP) [file pone.0314523.s005.zip › PACE Corrected/S2_Fig.tif]

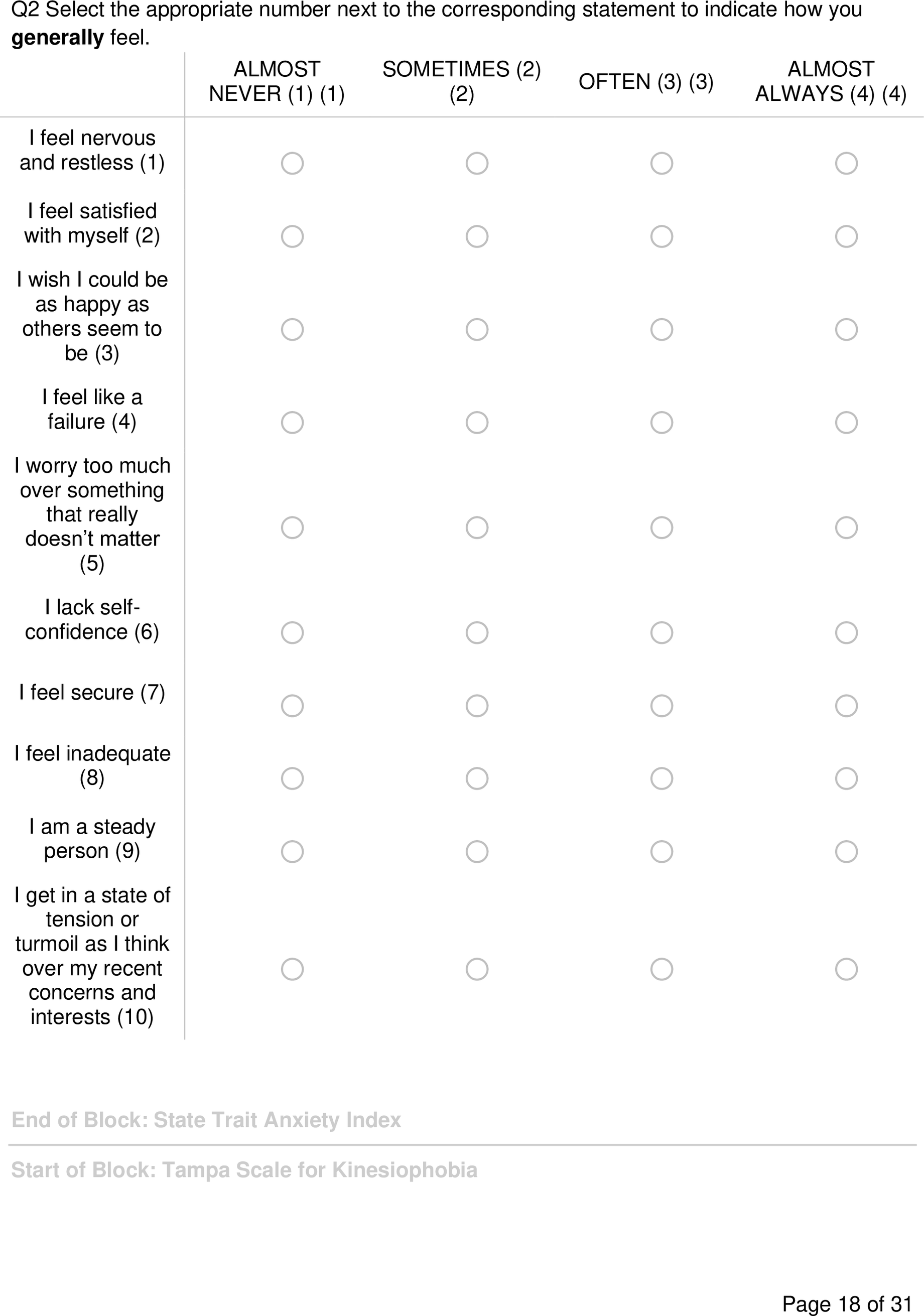

Supplement: S1 File — (ZIP) [file pone.0314523.s005.zip › PACE Corrected/S2_Fig.tif]

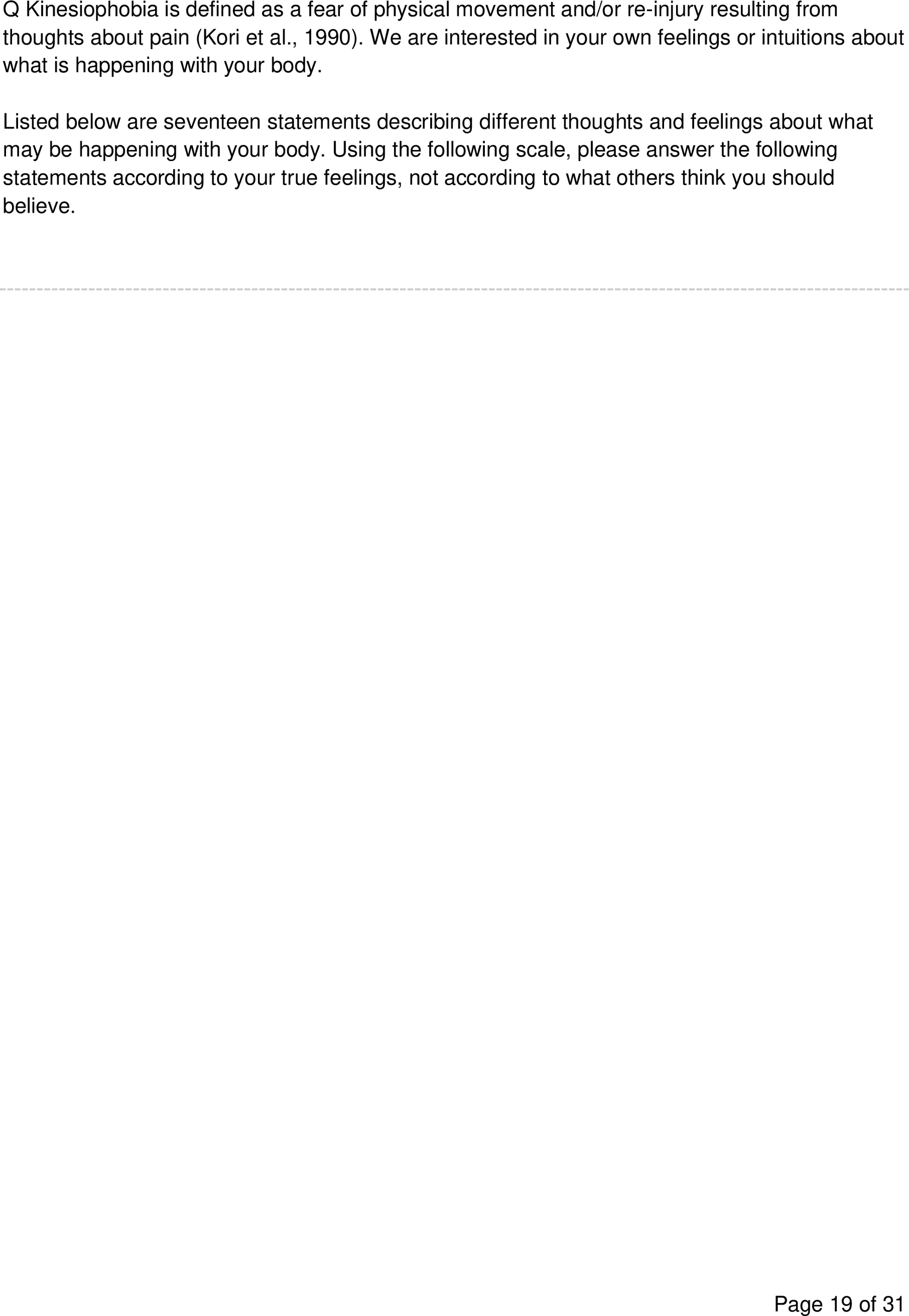

Supplement: S1 File — (ZIP) [file pone.0314523.s005.zip › PACE Corrected/S2_Fig.tif]

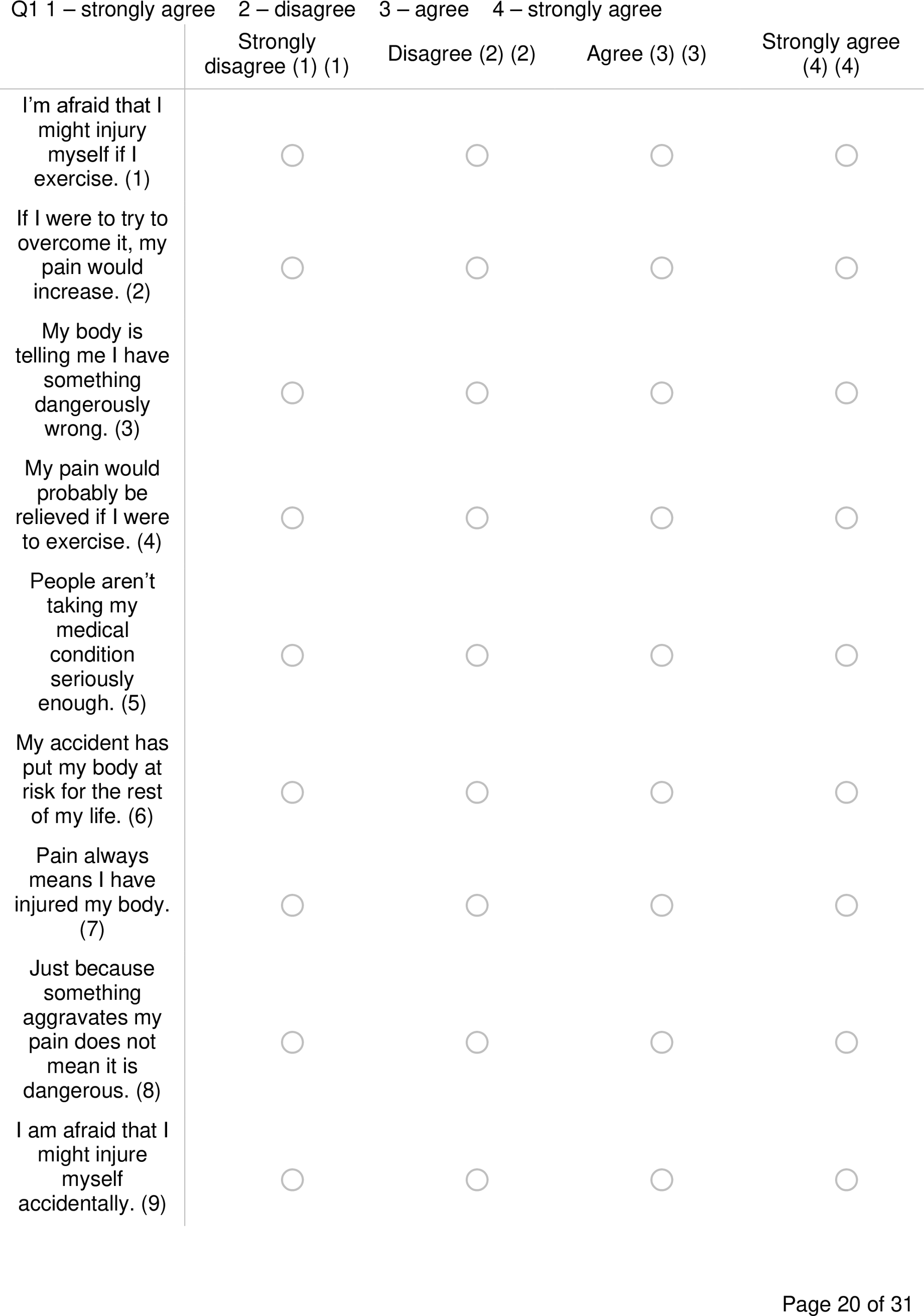

Supplement: S1 File — (ZIP) [file pone.0314523.s005.zip › PACE Corrected/S2_Fig.tif]

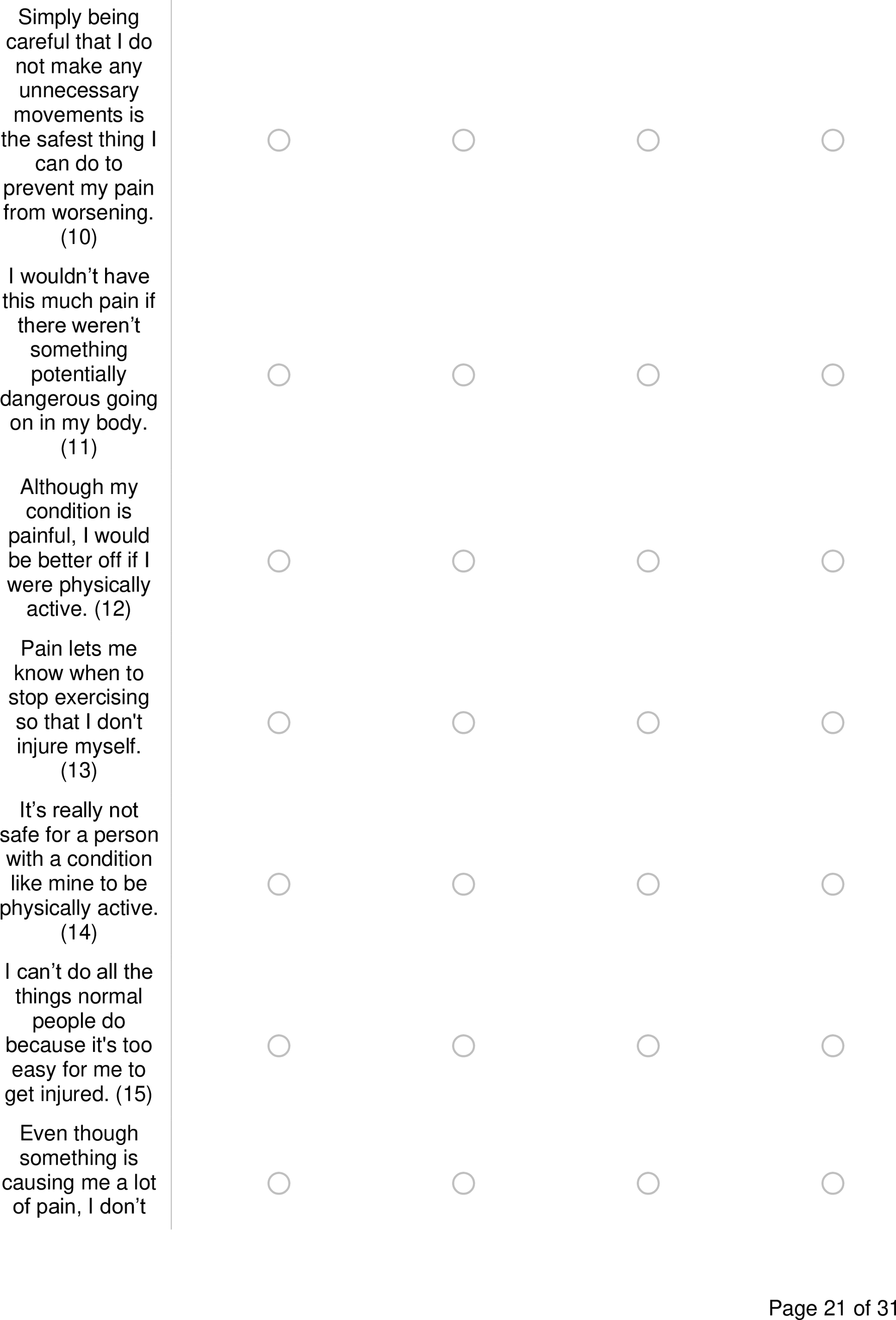

Supplement: S1 File — (ZIP) [file pone.0314523.s005.zip › PACE Corrected/S2_Fig.tif]

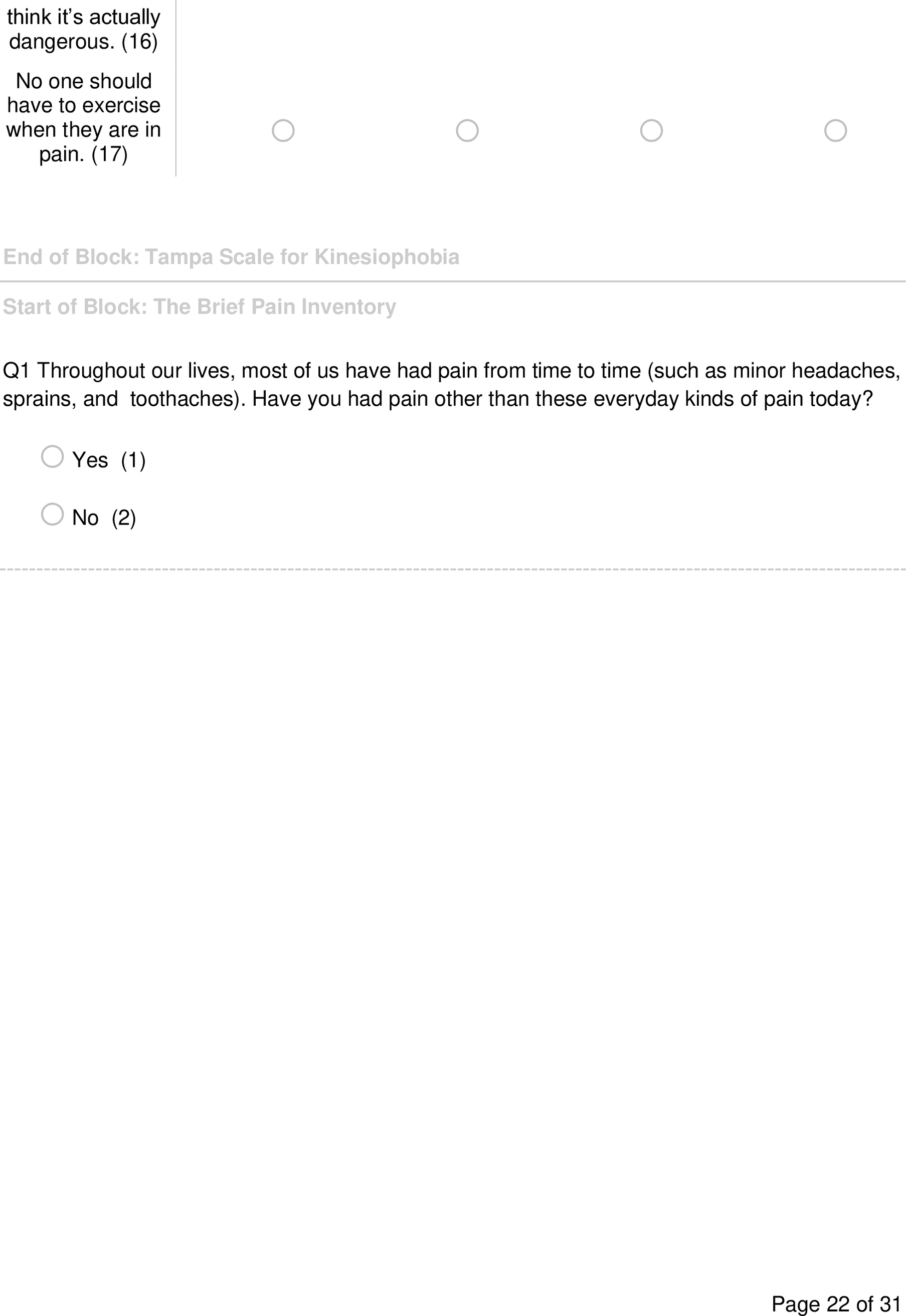

Supplement: S1 File — (ZIP) [file pone.0314523.s005.zip › PACE Corrected/S2_Fig.tif]

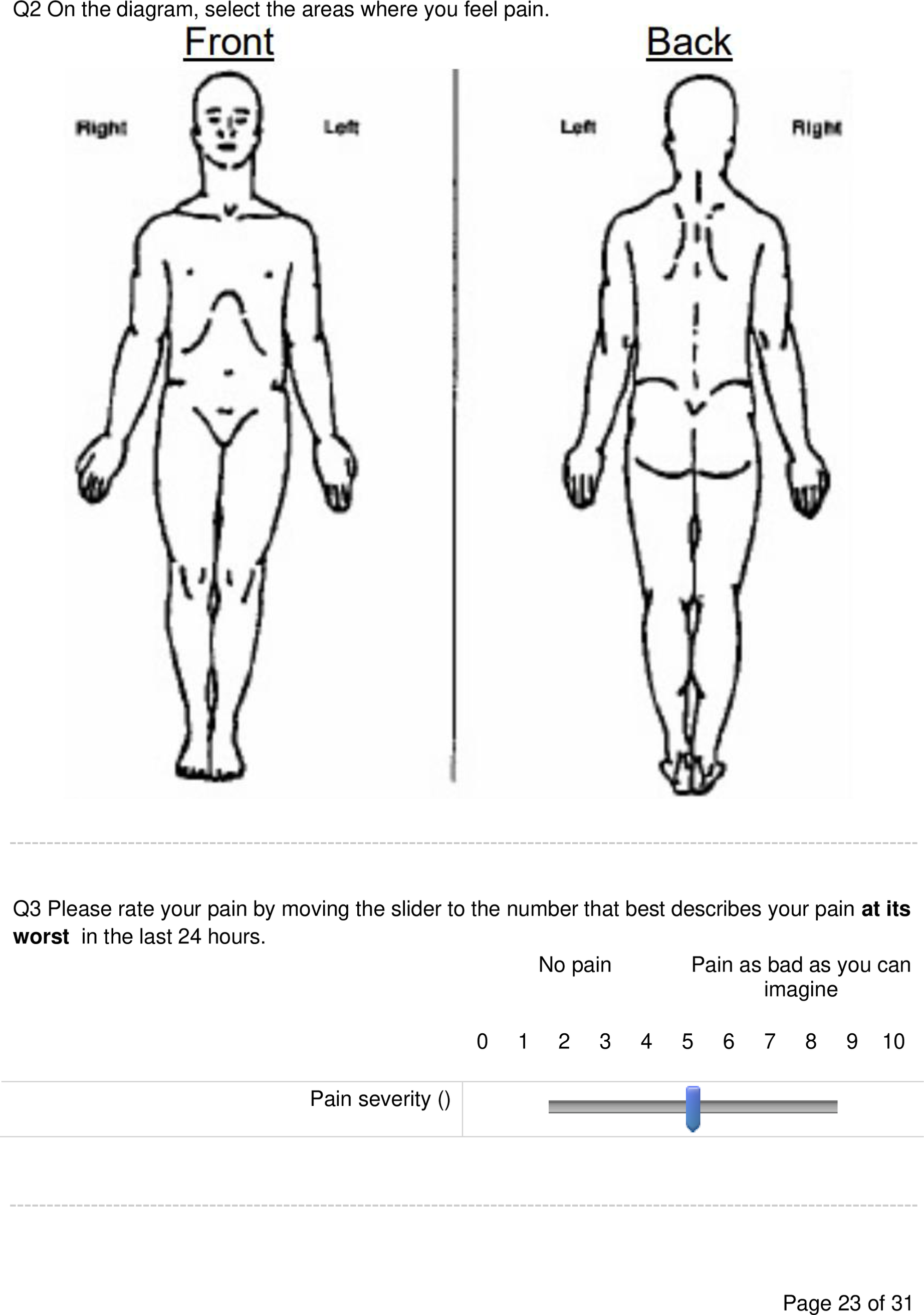

Supplement: S1 File — (ZIP) [file pone.0314523.s005.zip › PACE Corrected/S2_Fig.tif]

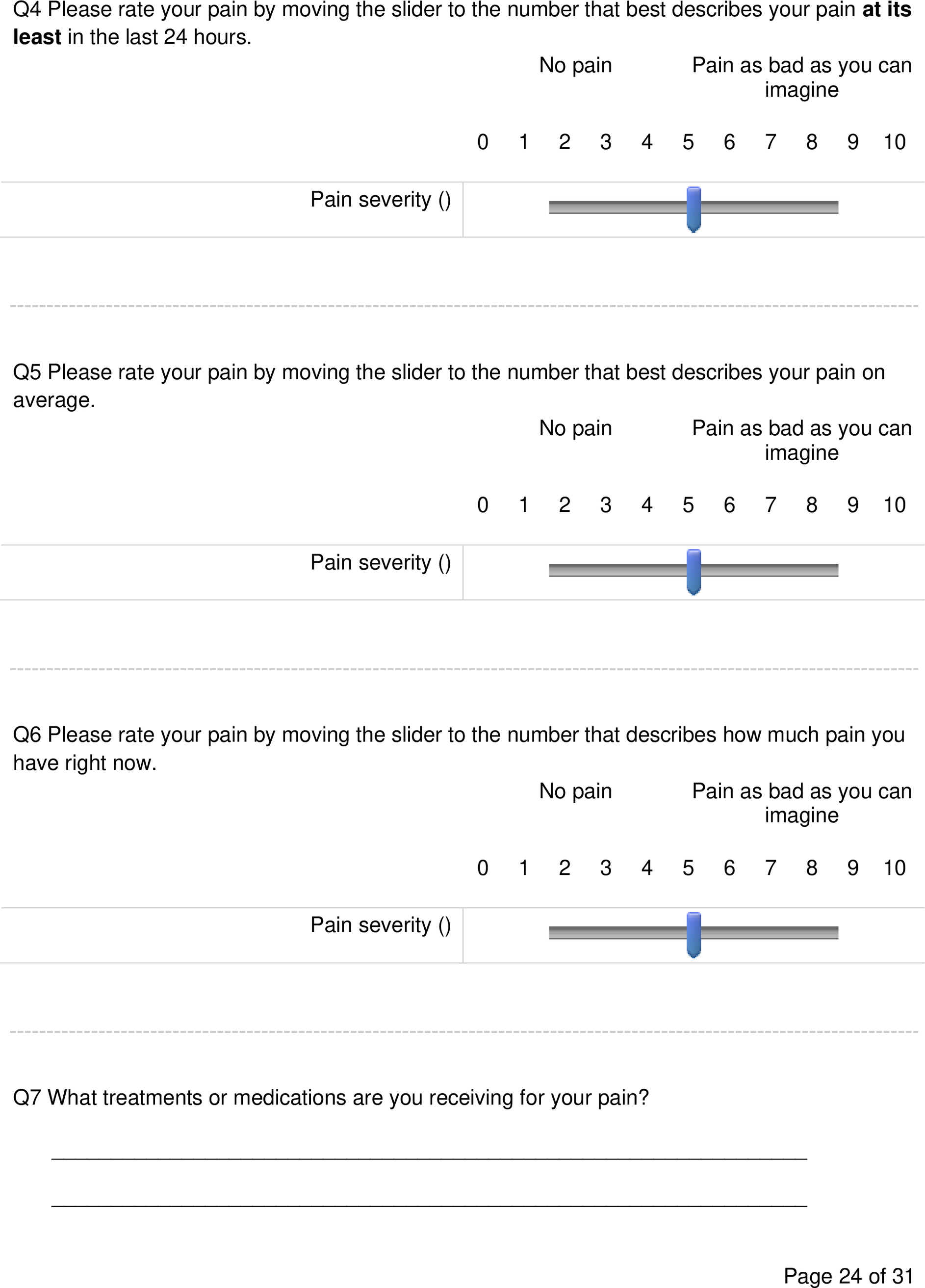

Supplement: S1 File — (ZIP) [file pone.0314523.s005.zip › PACE Corrected/S2_Fig.tif]

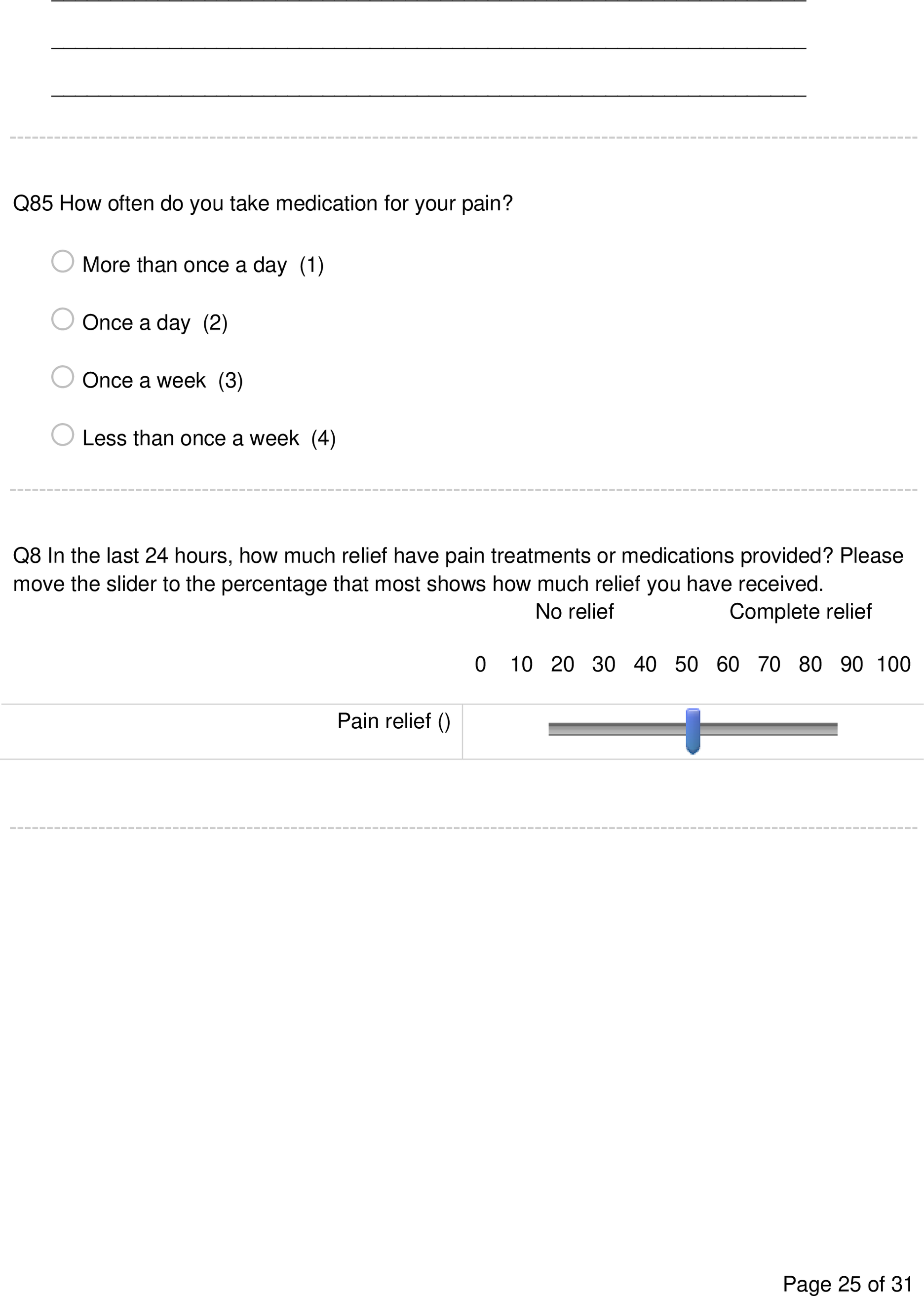

Supplement: S1 File — (ZIP) [file pone.0314523.s005.zip › PACE Corrected/S2_Fig.tif]

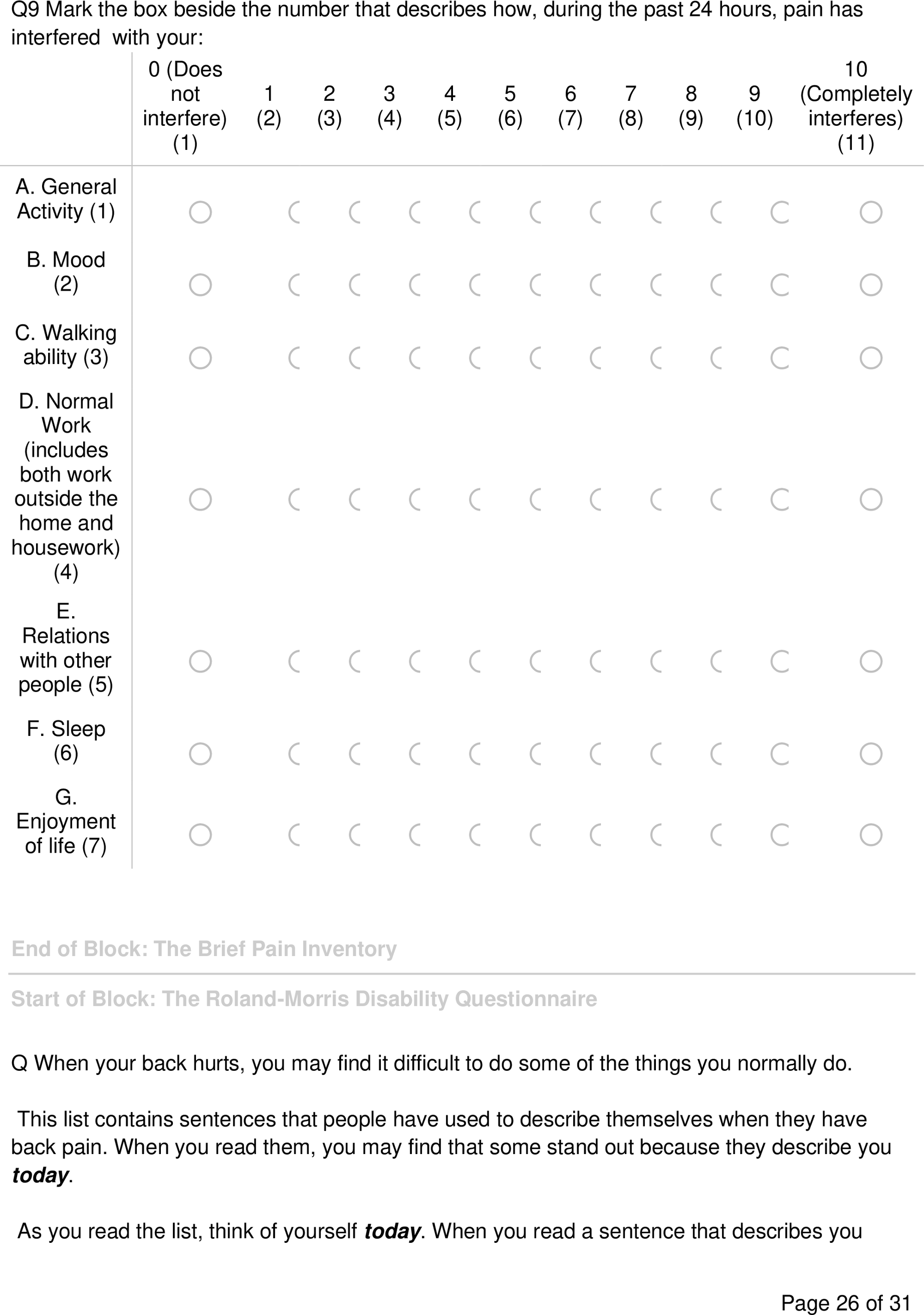

Supplement: S1 File — (ZIP) [file pone.0314523.s005.zip › PACE Corrected/S2_Fig.tif]

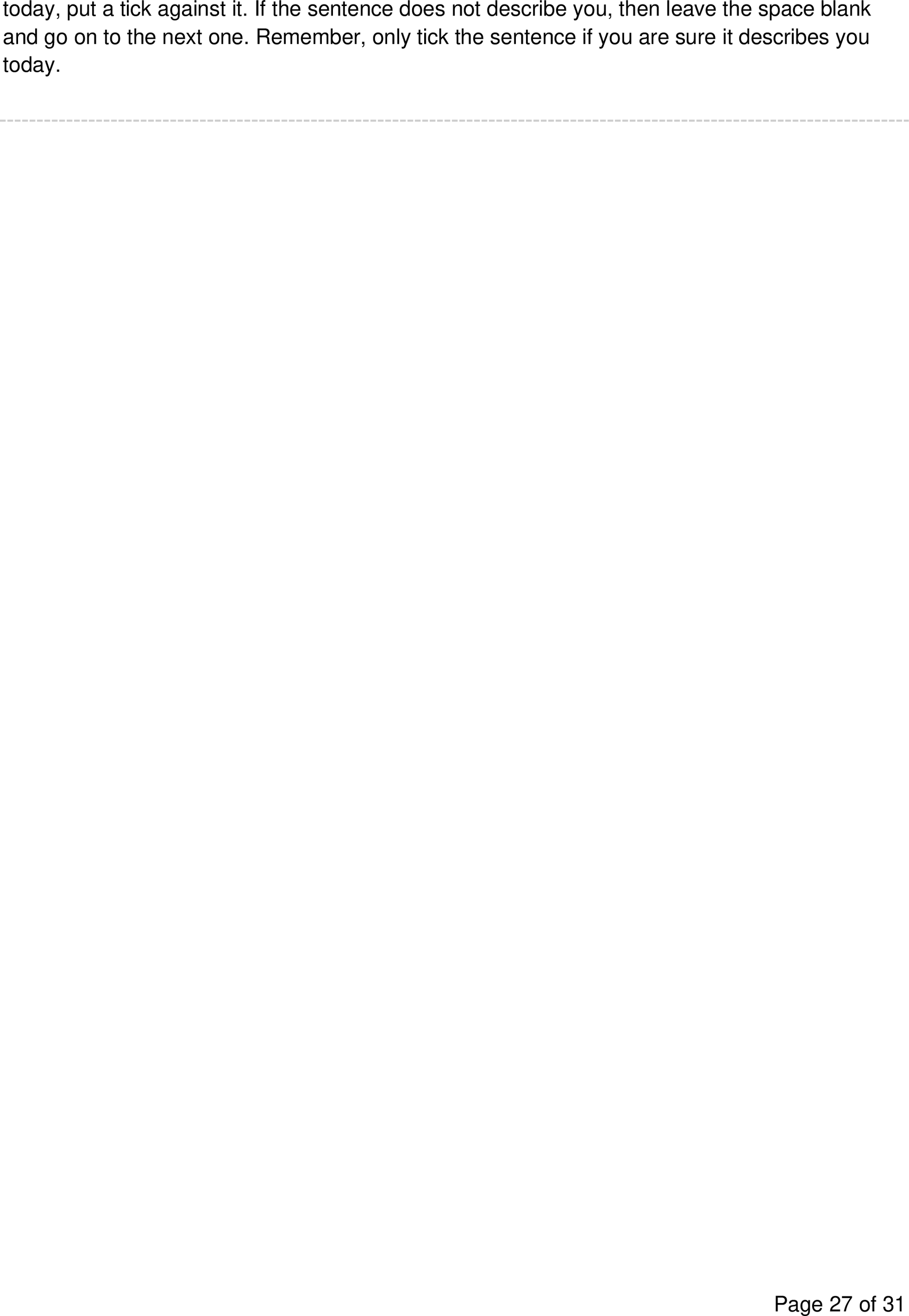

Supplement: S1 File — (ZIP) [file pone.0314523.s005.zip › PACE Corrected/S2_Fig.tif]

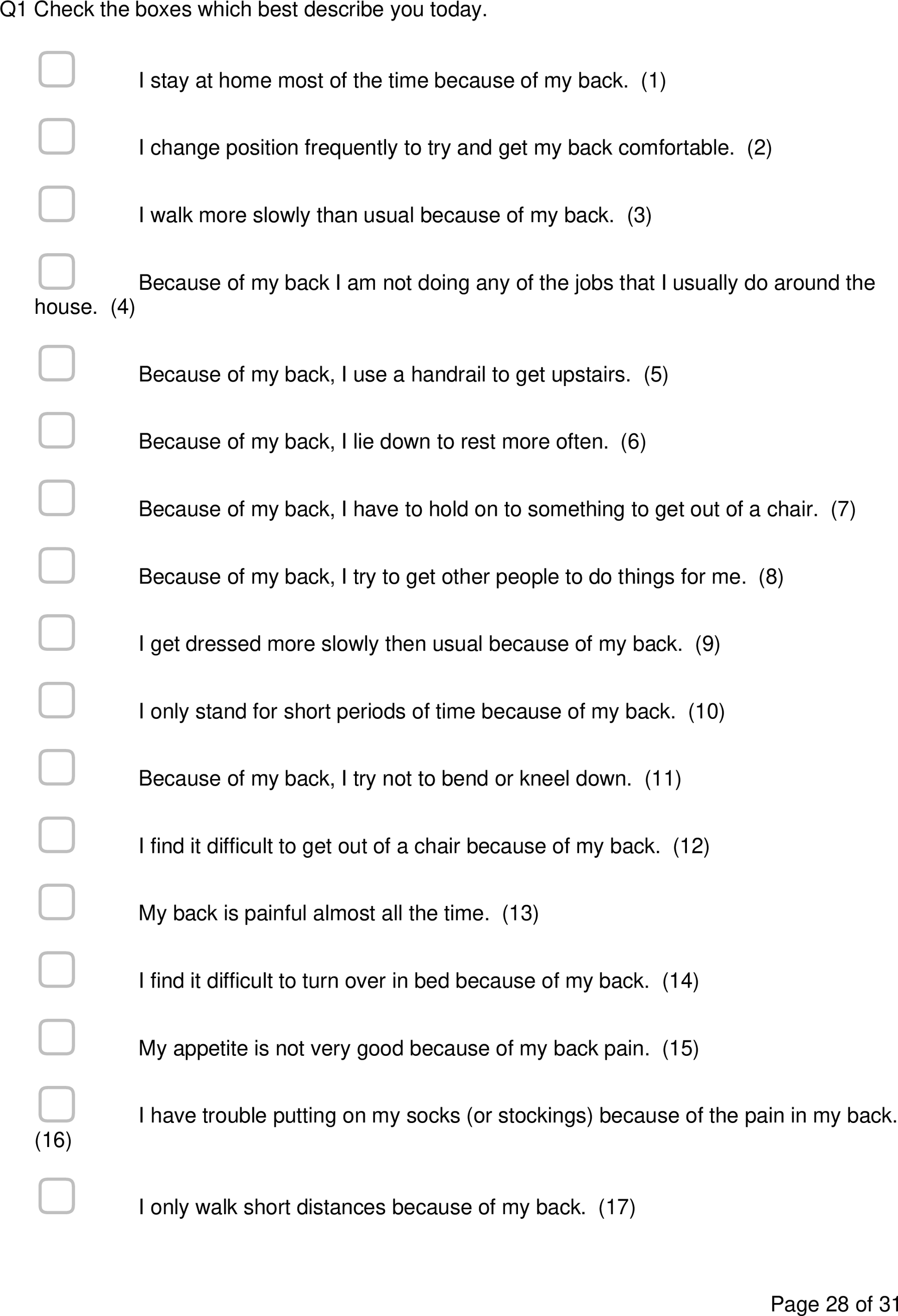

Supplement: S1 File — (ZIP) [file pone.0314523.s005.zip › PACE Corrected/S2_Fig.tif]

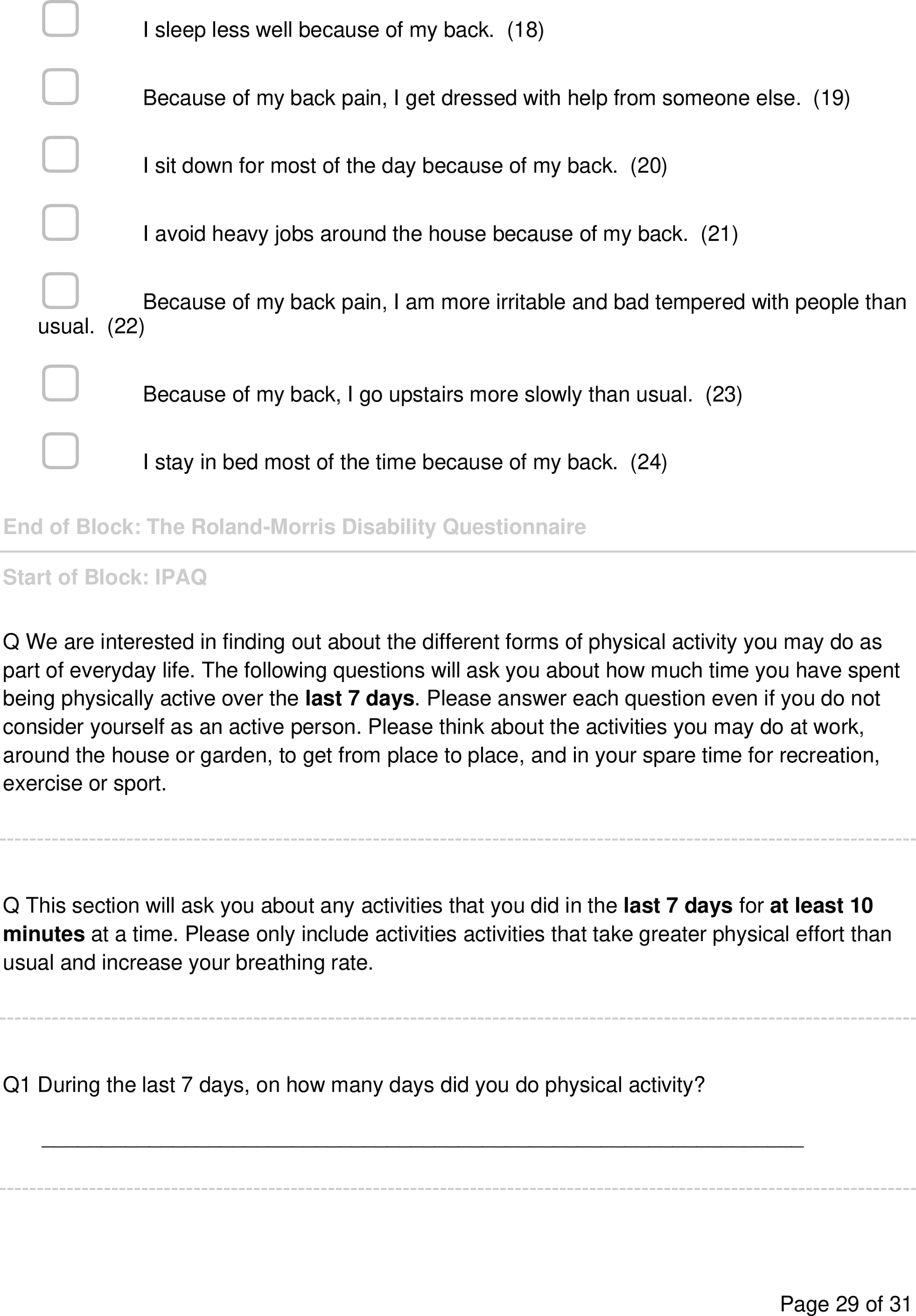

Supplement: S1 File — (ZIP) [file pone.0314523.s005.zip › PACE Corrected/S2_Fig.tif]

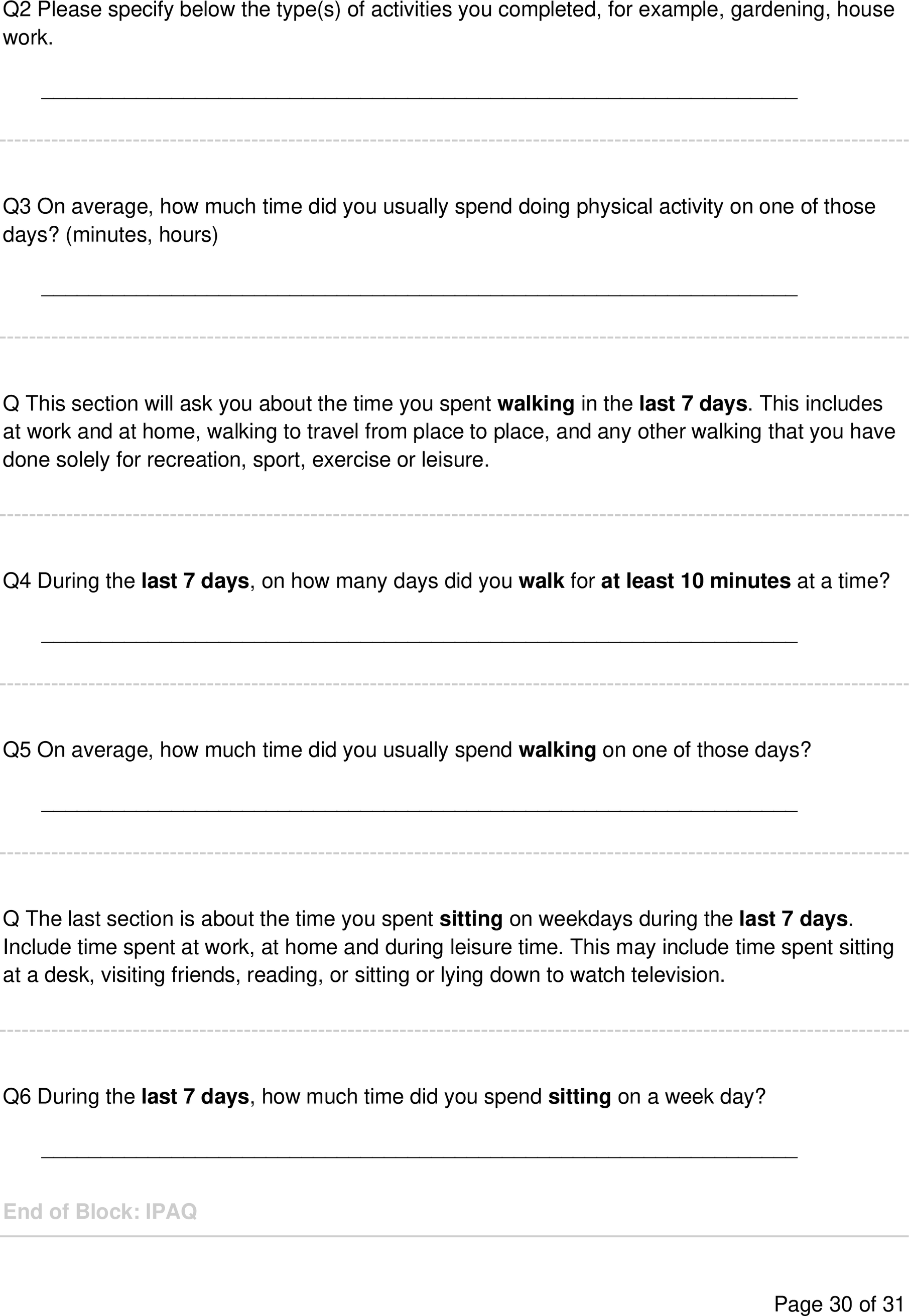

Supplement: S1 File — (ZIP) [file pone.0314523.s005.zip › PACE Corrected/S2_Fig.tif]

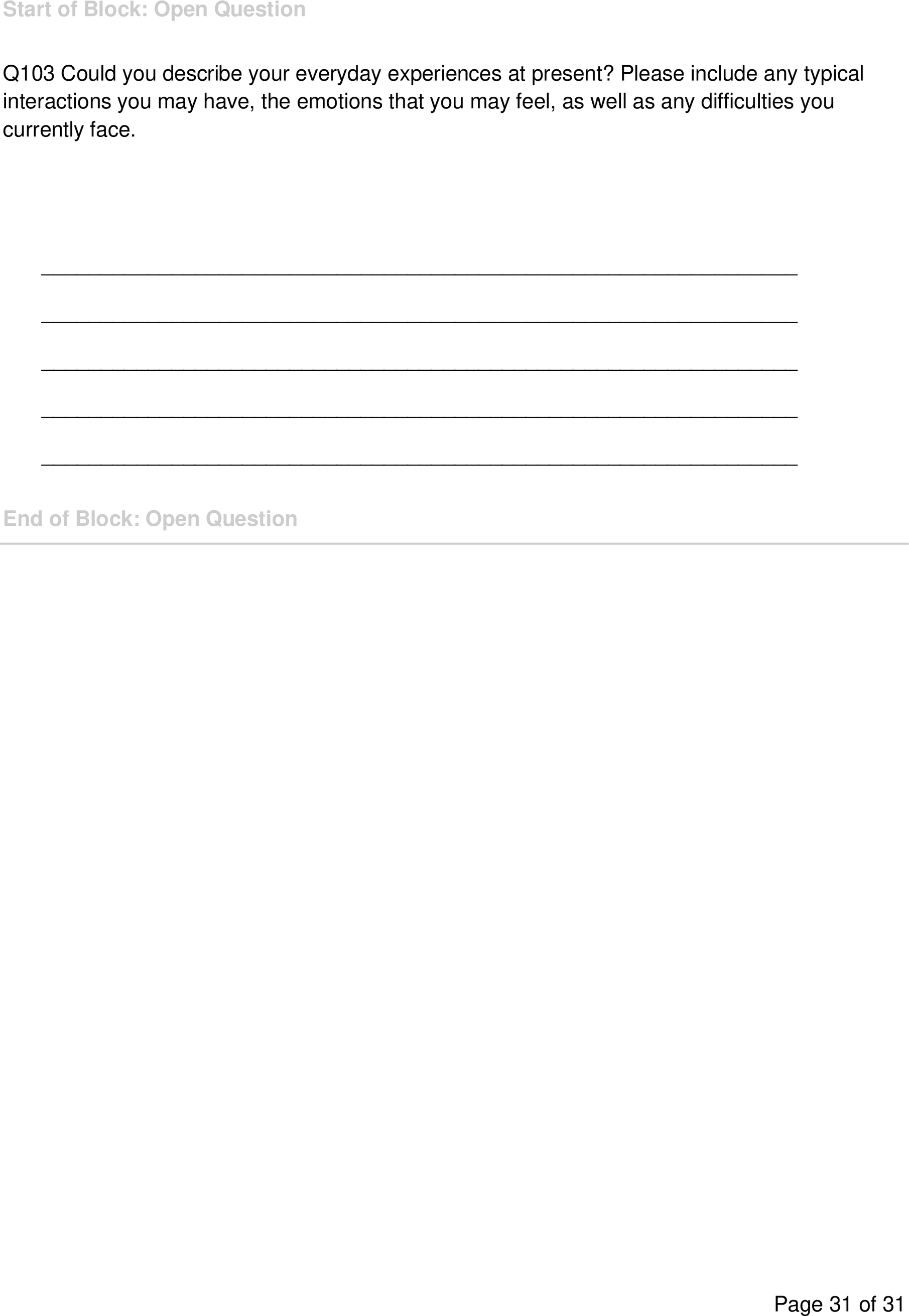

Supplement: S1 File — (ZIP) [file pone.0314523.s005.zip › PACE Corrected/S2_Fig.tif]
